# Ser46-Phosphorylated MARCKS Is a Marker of Neurite Degeneration at the Pre-aggregation Stage in PD/DLB Pathology

**DOI:** 10.1523/ENEURO.0217-18.2018

**Published:** 2018-09-04

**Authors:** Kyota Fujita, Hidenori Homma, Kanoh Kondo, Masashi Ikuno, Hodaka Yamakado, Kazuhiko Tagawa, Shigeo Murayama, Ryosuke Takahashi, Hitoshi Okazawa

**Affiliations:** 1 Department of Neuropathology, Medical Research Institute and Center for Brain Integration Research, Tokyo Medical and Dental University, 1-5-45 Yushima, Bunkyo-ku, Tokyo 113-8510, Japan; 2 Department of Neurology, Graduate School of Medicine, Kyoto University, Yoshida-Konoe-Cho, Sakyo-Ku, Kyoto, 606-8501, Japan; 3 Department of Neuropathology, Brain Bank for Aging Research, Tokyo Metropolitan Institute of Gerontology, 35-2, Sakae-Cho, Itabashi-ku, Tokyo 173-0015, Japan; 4 Center for Brain Integration Research, Tokyo Medical and Dental University, 1-5-45 Yushima, Bunkyo-ku, Tokyo 113-8510, Japan

**Keywords:** Biomarker, degeneration, MARCKS, neurite, phosphorylation

## Abstract

Phosphorylation of myristoylated alanine-rich C kinase substrate (MARCKS) reflects neurite degeneration at the early stage of Alzheimer’s disease (AD), before extracellular Aβ aggregates are histologically detectable. Here, we demonstrate that similar changes in MARCKS occur in Parkinson’s disease (PD) and dementia with Lewy bodies (DLB) pathologies in both mouse models and human patients. The increase in the level of pSer46-MARCKS began before α-synuclein aggregate formation, at a time when human α-Syn-BAC-Tg/GBA-hetero-KO mice exhibited no symptoms, and was sustained during aging, consistent with the pattern in human postmortem brains. The results strongly imply a common mechanism of pre-aggregation neurite degeneration in AD and PD/DLB pathologies.

## Visual Abstract

**Figure:**
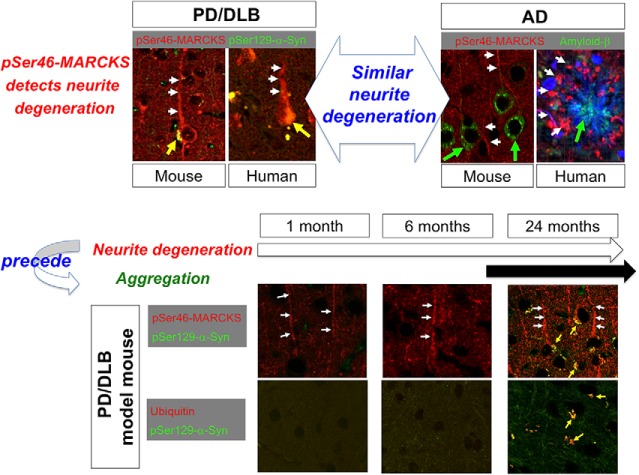

## Significance Statement

At early stages of Alzheimer’s disease, before the aggregation of extracellular Aβ, phosphorylation of MARCKS at Ser46 reflects neurite degeneration. In this study, we confirmed the similar changes in both mouse models and human patients of Parkinson’s disease (PD) and dementia with Lewy bodies (DLB). Phosphorylation of MARCKS at Ser46 was increased before α-synuclein aggregation was detected, and the increase of pSer46-MARCKS was sustained during aging. These results suggest that neurite degeneration detected by pSer46-MARCKS is a common pre-aggregation mechanism shared by AD and PD/DLB pathologies.

## Introduction

Protein aggregation is a widely accepted hallmark of neurodegenerative disorders, including Alzheimer’s disease (AD), Parkinson’s disease (PD), dementia with Lewy bodies (DLB), frontotemporal lobar degeneration (FTLD), Huntington’s disease (HD), spinocerebellar ataxia (SCA), and amyotrophic lateral sclerosis (ALS). In general, the disease-related proteins are assumed to adopt misfolded structures that are subsequently converted to aggregation-prone structures, including β-sheets. However, the details of chronological and/or stochastic changes in these structures remain largely unknown, triggering debates about the identities of the true toxic species, as well as whether aggregated or soluble proteins are toxic.

Clinical trials of AD therapeutics have had a considerable impact on these discussions. Passive immunization with anti-Aβ antibodies has been largely successful in decreasing the abundance of extracellular Aβ plaques in the brain. However, those trials reported discrepancies between improvements in Aβ-PET and amelioration of clinical symptoms (https://www.alzforum.org/news/research-news/pib-pet-biomarker-study-confirms-bapineuzumab-lowers-amyloid). Therefore, elucidation of the early-stage pathologies is now an urgent issue for understanding pathogenesis and developing therapeutics of AD, and the situation is largely similar in the other neurodegenerative diseases including PD/DLB.

Comprehensive phosphoproteomic analysis of brain samples from mouse AD models and human AD patients revealed that changes in the phosphorylation of some proteins are initiated before Aβ plaques are histologically detectable (Tagawa et al., 2015). In one such protein, MARCKS, phosphorylation at Ser46 can be detected in degenerated neurites of mice at the age of 1 month, before cognitive symptoms and Aβ plaques arise (Tagawa et al., 2015). Phosphorylation of MARCKS at Ser46 is triggered by damage-associated molecular patterns (DAMPs), especially by HMGB1, via TLR4 (Fujita et al., 2016).

In this study, we detected similar phosphorylation of MARCKS at Ser46 in BAC-Tg mice overexpressing human normal α-synuclein (α-Syn) in the glucocerebrosidase (GBA)-heterozygous-knockout (KO) background (human α-Syn-BAC-Tg/GBA-hetero-KO mice), as well as in human DLB patients. The histologic features of phosphorylation of MARCKS on serine 46 (pSer46-MARCKS) and chronological progression in the brain were similar in AD and PD/DLB. Intriguingly, this marker of neurodegeneration became positive before formation of histologically detectable α-Syn aggregates. These results cast light on the relationship between neuronal degeneration and protein aggregation, and reveal that the initiation of neurite degeneration precedes formation of protein aggregates.

## Materials and methods

### Mouse PD/DLB model

Normal human α-Syn-BAC-Tg mice were created as previously described (Yamakado et al., 2012). Briefly, the BAC-Tg construct (PAC AF163864 and BAC AC097478, containing 28 kb of the 5′-flanking sequences and 50 kb of the 3′-flanking sequences, in addition to the entire human gene) was microinjected into C57BL6/J ova, yielding homozygous α-Syn-Tg mice. GBA-hetero-KO mice were purchased from the Jackson Laboratory (B6.129S6-Gbatm1Nsb/J, stock no. 003321) and mated with human α-Syn-BAC-Tg mice. The resultant normal human α-Syn-BAC-Tg/GBA-hetero-KO (homo/hetero) mice were maintained as a line. The α-Syn-BAC-Tg/GBA-hetero-KO mice were crossed for >10 generations, and 3 male α-Syn-BAC-Tg/GBA-hetero-KO mice were used at the ages of 1, 6, and 24 months for immunohistochemistry and biological analysis. Analysis of detailed phenotypes of α-Syn-BAC-Tg/GBA-hetero-KO mice will be reported elsewhere by one of the authors (R.T.).

## Immunohistochemistry

For immunohistochemistry, mouse (three males at each time point) or human (five females in each group) brains were fixed with 4% paraformaldehyde and embedded in paraffin. The paraffin-embedded brain sections were deparaffinized, rehydrated, antigen-activated (microwaved in 10 mm citrate buffer, pH 6.0, for 5 min at 100°C; this process was repeated three times), and cooled to room temperature (RT). For staining for phospho-α-Syn, the sections were additively activated by 98% formic acid (Wako, 066-00461) for 5 min at RT. The sections were washed twice for 5 min each with PBST (PBS containing 0.1% Tween 20), treated with PBS containing 0.5% Triton X-100 for 20 min, and washed three times with PBST for 5 min. After blocking (10% FBS for 30 min at 37°C), the sections were incubated sequentially with PBS containing 2% FBS and 0.1% Triton X-100, primary antibodies [mouse anti–phospho-α-Syn (Ser129; 1:2000, Wako, 015-25191) for 60 min at 37°C; mouse anti–α-Syn (1:1000, Abcam, ab27766) for 12 h at 4°C; rabbit anti–phospho-MARCKS (Ser46; 1:1000, GL Biochem) for 120 min at 37°C; or mouse anti-ubiquitin antibody (1:1000, Cell Signaling Technology, 3936S); rabbit anti–phospho-ERK1/2 detecting mouse Thr203/Tyr205-ERK1/Thr183/Tyr185-ERK2 and human Thr202/Tyr204-ERK1/Thr185/Tyr187-ERK2 (1:250, Cell Signaling Technology, 4370), mouse anti-MAP2 (1:100, Santa Cruz, sc-32791), or mouse anti-GFAP (1:2000, Sigma, C9205) for 12 h at 4°C], and secondary antibodies [Alexa Fluor 488–labeled anti-mouse IgG (1:1000, Invitrogen) or Cy3-labeled anti-rabbit IgG (1:500, Jackson ImmunoResearch) for 60 min at RT]. pSer46-MARCKS antibody was generated, and the quality was examined, as reported previously (Fujita et al., 2016). For double-labeling of pSer129-α-Syn and ubiquitin, anti–pSer129-α-Syn antibody was labeled with the Zenon Alexa Fluor 488 mouse IgG1 labeling kit (Z-25002, Invitrogen). Nuclei were stained with DAPI (Dojindo Laboratories, D523). Images were acquired by confocal microscopy: Olympus FV1200 IX83.

### Western blotting

Mouse cerebral cortex and human temporal lobe tissues were dissolved in extraction buffer containing 2% SDS, 1 mm DTT, and 10 mm Tris-HCl (pH 7.5) and homogenized using 20 strokes of a Dounce glass homogenizer on ice. The crude extracts were centrifuged at 16,000 × *g* at 4°C for 10 min, added to an equal volume of sample buffer (0.1 m Tris-HCl, pH 7.5, 4% SDS, 20% glycerol, 12% β-mercaptoethanol, and 1% bromophenol blue), and boiled at 95°C for 10 min.

Samples were separated by SDS-PAGE, transferred onto polyvinylidene difluoride membranes (Immobilon-P, Merck Millipore) using the semi-dry method, blocked with 5% milk or 2% BSA in TBST (10 mm Tris/HCl, pH 8.0, 150 mm NaCl, and 0.05% Tween 20), and reacted with primary and secondary antibodies diluted in Can Get Signal solution (Toyobo). Primary and secondary antibodies were diluted as follows: mouse anti–phospho-α-Syn (Ser129; 1:5000, Wako, 015-25191); mouse anti–α-Syn (1:5000, Abcam, ab27766) for 12 h at 4°C; rabbit anti–phospho-MARCKS (Ser46; 1:200,000, GL Biochem) for 120 min at 37°C; mouse anti-ubiquitin antibody (1:5000, Cell Signaling Technology, 3936S) for 12 h at 4°C; rabbit anti–phospho-ERK1/2 (Thr203/Tyr205(mouse)-ERK1/Thr183/Tyr185(mouse)-ERK2, Thr202/Tyr204(human)-ERK1/Thr185/Tyr187(human)-ERK2; 1:10,000, Cell Signaling Technology, 4370); or rabbit anti-ERK1/2 (1:5000, Cell Signaling Technology, 4695S); HRP-conjugated anti-mouse IgG (1:3000, GE Healthcare, NA931VA) and anti-rabbit IgG (1:3000, GE Healthcare, NA934VS). ECL prime (GE Healthcare, RPN2232) or ECL select (GE Healthcare, RPN2235) was used to detect the bands using LAS4000 (GE Healthcare).

## Immunoprecipitation

Mouse and human brain samples were lysed with TNE buffer (10 mm Tris-HCl, pH 7.5, 10 mm NaCl, 1 mm EDTA, 1% NP-40, 0.5% protease inhibitor cocktail, 0.5% phosphatase inhibitor cocktail). Aliquots were incubated with a 50% slurry of protein G Sepharose beads (GE Healthcare), followed by 3-min centrifugation (2000 × *g*). The supernatants were incubated with 1 μg rabbit anti–pSer46-MARCKS or mouse anti–pSer129-α-synuclein antibody overnight at 4°C; incubated with protein G Sepharose beads (GE Healthcare) for 4 h; washed with TNE buffer; and eluted with sample buffer.

### Peripheral blood cell collection from AD model mice

Peripheral blood cells [polymorphonuclear leukocytes (PMNs) and mononuclear cells (MCs)] were collected using Polymorphprep solution (Alere Technologies) according to the manufacturer’s protocol. Briefly, 1 ml venous blood with EDTA (final concentration 2.0 mm) was carefully layered over 1 ml Polymorphprep in 15-ml tubes. After 30-min centrifugation at 500 × *g* at room temperature, plasma was removed, and the layer containing PMNs and MCs was obtained. Collected aliquots were diluted with 0.45% NaCl, and cell pellets were obtained by centrifugation at 400 × *g* for 10 min. Pellets were lysed with lysis buffer (10 mm Tris-HCl, pH 7.5, 0.2% SDS, 0.5% protease inhibitor cocktail, and 0.5% phosphatase inhibitor cocktail), added with sample buffer containing 62.5 mm Tris-HCl, pH 6.8, 2% (w/v) SDS, 2.5% (v/v) 2-mercaptoethanol, 5% (v/v) glycerol, and 0.0025% (w/v) bromophenol blue, and subjected to SDS-PAGE.

### Mass analysis

For phosphoproteomic analysis, phosphorylated peptides were prepared as described previously (Tagawa et al., 2015; Fujita et al., 2016). Briefly, cerebral cortex tissues were dissected from the temporal pole or occipital pole of postmortem brains of five females in each group of AD and DLB, their extracts were denatured by detergent and heat treatment, and cysteine residues were reduced and blocked by alkylation. Protein samples were digested with trypsin. Phosphopeptides were enriched using the Titansphere Phos-TiO Kit (GL Sciences), labeled using an iTRAQ Reagent multiplex kit (SCIEX), and fractionated by strong cation exchange chromatography. Each fraction was analyzed using a LC-MS/MS system (Triple TOF 5600 System, Eksigent LC system; SCIEX) and a C18 column (0.1 × 100 mm; KYA Technologies Corporation). The ion spray voltage was 2.3 kV, and the information-dependent acquisition (IDA) setting was 400–1250 *m*/*z*.

### Data analysis

Mass spectrum data of peptides were acquired and analyzed by Analyst TF (version 1.6; AB SCIEX). Proteins corresponding to the results were identified by searching UniProtKB/Swiss-Prot (downloaded from http://www.uniprot.org on June 22, 2010) and identified by ProteinPilot (version 4; AB SCIEX), which employs the Paragon algorithm (Shilov et al., 2007). Tolerance for the search of peptides by ProteinPilot was set to 0.05 Da for MS and 0.10 Da for MS/MS analyses. Redundantly identified proteins were excluded using the Pro Group algorithm (AB SCIEX). The confidence scores of protein or peptide identification were calculated by ProteinPilot and used as the confidence threshold. The threshold for detection was set at 95% confidence, and peptides with >95% confidence were accepted as identified peptides.

Quantification of proteins was performed through analysis of iTRAQ reporter groups in the MS/MS spectrum that were generated on fragmentation in the mass spectrometer. For quantification of peptides and proteins, bias correction was employed to normalize signals among different iTRAQ reporters under the assumption that the total amount of signal from each iTRAQ should be equal. For quantification of peptides, the bias correction option was used to normalize different iTRAQ signals.

Peptide ratio was calculated as the ratio of reporter signals in patient versus disease control samples after bias correction. The details of this formulation are provided in the manual from AB SCIEX.

The results of the peptide summary from ProteinPilot were output as an Excel file for further data analysis. Quantity of a peptide fragment was calculated as the geometric mean of signal intensities of multiple MS/MS fragments including the phosphorylation site. For every disease group, biological differences relative to the control group were evaluated by Welch’s test. For multiple-testing correction, *p*-values were adjusted using the Benjamini–Hochberg procedure. Phosphopeptides that changed in multiple disease groups were identified and selected for further analysis.

### Human brain

Temporal- and occipital-pole human brain samples used for proteome analysis were dissected from five female AD, five female DLB, and five female age-matched normal control patients and deep-frozen (–80°C) within 1 h after death.

### Ethics

The experiments with human samples were approved by the Ethics Committees of the Tokyo Medical and Dental University (O2014-005-03). All animal experiments were approved by the Institutional Animal Care and Use Committee of Tokyo Medical and Dental University (A2017-242A). The same experiments were also approved by the Committees on Gene Recombination Experiments of Tokyo Medical and Dental University (2016-007C4).

## Results

### Elevated levels of pSer46-MARCKS in the phosphoproteomes of human AD and DLB

**Figure 1.**
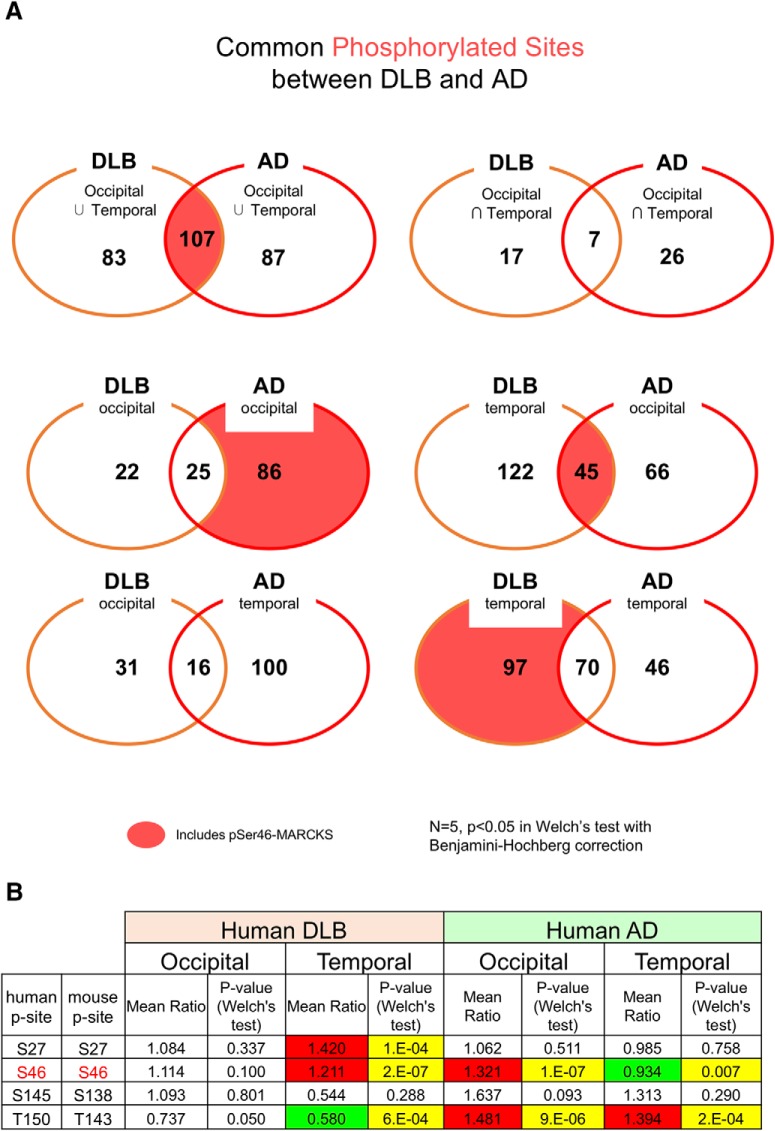
Comprehensive phosphoproteomic analyses of postmortem human AD and DLB brains. ***A***, Occipital and temporal tips of pathologically pure AD (five females) and DLB (5 females) brains along with age-matched normal controls (5 females) were used for comprehensive phosphoproteome analyses as described previously (Tagawa et al., 2015). Independent phospho-sites were compared between AD and DLB. ***B***, Comparison of changes at four different phosphorylation sites in MARCKS [Ser27/Ser27, Ser46/Ser46, Ser145/Ser138, and Thr150/Thr143 (human/mouse)] between human DLB and AD patients. (See also Fig. 1-1.)

Following previous analyses of brain samples from AD mouse models and human AD patients (Tagawa et al., 2015), we performed comprehensive phosphoproteome analysis with postmortem human DLB patients. In this case, we selected postmortem brains with pure pathology, with intracellular α-Syn aggregates in neurons but without extracellular Aβ aggregates or cytoplasmic tau/TDP43 pathology. The comparison revealed that 107 phosphorylation sites were shared between AD and DLB samples. One such modification, pSer46-MARCKS, was elevated in both AD mouse models and postmortem human AD brains (Fujita et al., 2016; Figs. 1*A* and 1-1). Interestingly, the pSer46-MARCKS level was elevated in the temporal but not the occipital lobe in DLB, but in the opposite pattern (i.e., occipital but not temporal lobe) in AD (Fig. 1*B*). In 5xFAD mice, the increase in the pSer46-MARCKS level at the early stage of the disease (preclinical/pre-aggregation phase) had normalized by the late stage (Fujita et al., 2016). Therefore, the discrepancy in pSer46-MARCKS levels between the two lobes might reflect the lobar dominance of neurodegeneration of each disease. Mass spectrometry results suggesting that common changes occurred in AD and PD/DLB human brains prompted us to investigate whether pSer46-MARCKS also reflects the preclinical/pre-aggregation phase in PD/DLB.

10.1523/ENEURO.0217-18.2018.f1-1 Figure 1-1 Detailed list of phosphorylated proteins in human AD and DLB cortex Download Figure 1-1, DOCX file.

### Increased pSer46-MARCKS in immunohistochemistry of human DLB

**Figure 2.**
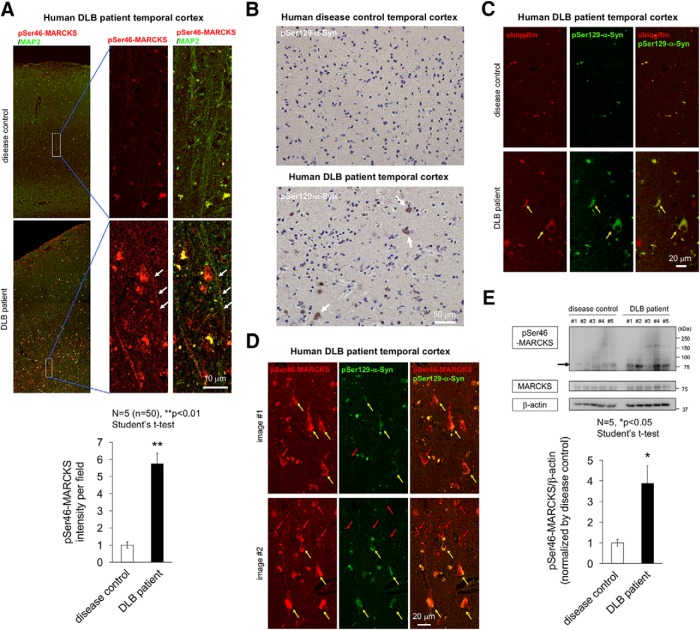
Immunohistochemistry of human DLB brains. ***A***, Costaining of pSer46-MARCKS with MAP2. Low and high magnification of temporal lobes from non-neurologic disease control patients (5 females) and human DLB patients (5 females). Images were acquired on an Olympus FV1200 IX83 confocal microscopy. All bar graphs indicate averages and SEM. In each patient, quantitative analyses of signal intensities (mean pixel intensities) were performed in 10 visual fields (100 × 100 μm) randomly selected from the corresponding area. Statistical analyses were performed with Student’s *t* test; **, *p* < 0.01. ***B***, Staining of pSer129-α-Syn revealed multiple cytoplasmic inclusions (Lewy bodies) in the same patient group. ***C***, Costaining of ubiquitin with pSer129-α-Syn. ***D***, Costaining of pSer46-MARCKS with pSer129-α-Syn. ***E***, Western blotting analysis of temporal lobes from non-DLB control patients and DLB patients with antibody against pSer46-MARCKS, total MARCKS, and β-actin. Graph shows the quantitative result of pSer46-MARCKS from 5 patients and 5 non-neurologic disease controls. The band intensity was normalized against β-actin. Statistical analyses were performed with Student’s *t* test; *, *p* < 0.05.

Next, we investigated whether pSer46-MARCKS was elevated in human postmortem DLB brains. For this purpose, we stained temporal lobe samples from DLB patients and found that apical dendrites marked with MAP2 were costained by pSer46-MARCKS (Fig. 2*A*). Meanwhile, we confirmed that this brain region included pSer129-α-Syn cytoplasmic inclusion bodies (Fig. 2*B*), which were mostly ubiquitinated (Fig. 2*C*). Because cytoplasmic staining of pSer46-MARCKS seemed like aggregates (Fig. 2*A*), we tested whether they were α-Syn aggregates and found that half of the cytoplasmic pSer46-MARCKS–positive structures in neurons were stained with pSer129-α-Syn (Fig. 2*D*, yellow arrow); however, the other half were not stained strongly with pSer129-α-Syn (Fig. 2*D*, red arrow). The pSer46-MARCKS–positive/pSer129-α-Syn–negative cells may correspond to surrounding neurons indirectly affected by HMGB1 released from damaged neurons that accumulates pSer129-α-Syn, suggesting that pathology continues to progress in human brain even at the terminal stage of DLB.

To support the above results of immunohistochemistry, we performed Western blotting of pSer46-MARCKS and total MARCKS with temporal lobe of human postmortem DLB patients and non-DLB control patients (Fig. 2*E*). The level of pSer46-MARCKS was increased in DLB patients.

### Increased pSer46-MARCKS in humanized α-Syn-Tg mice

**Figure 3.**
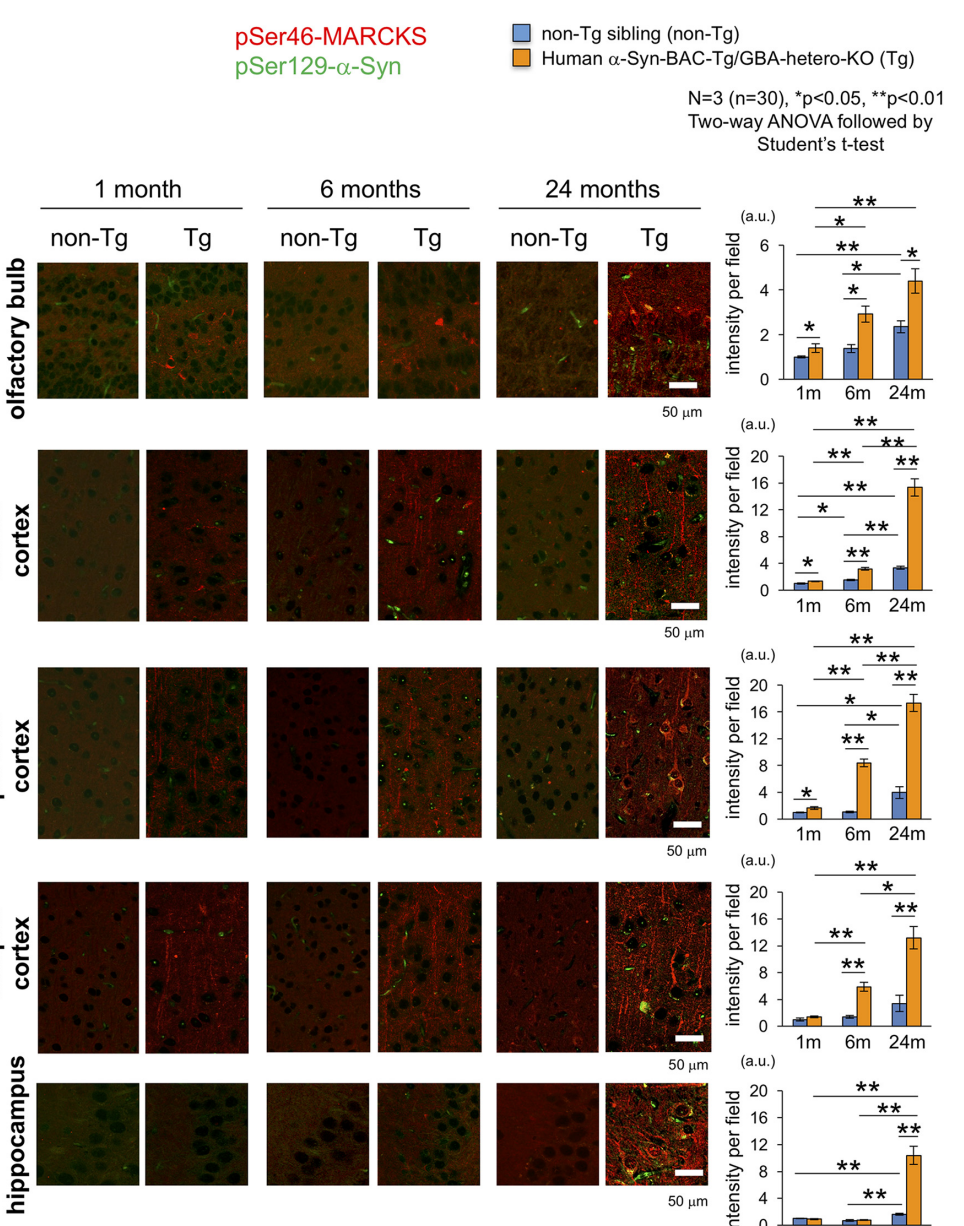
Chronological changes of pSer46-MARCKS in human α-Syn-BAC-Tg/GBA-hetero-KO mice. pSer46-MARCKS and pSer129-α-Syn were costained in human normal α-Syn-BAC-Tg/glucocerebrosidase (GBA)-hetero-KO mice at 1, 6, and 24 months of age (3 male mice at each time point). Images were acquired by Olympus FV1200 IX83 confocal microscopy. All bar graphs show average and SEM. Three mice were used for each group, and quantitative analyses of signal intensities (mean pixel intensities) were performed in 10 visual fields (100 × 100 μm) of each mouse, randomly selected from the brain region. Statistical analyses were performed with two-way ANOVA followed by Student’s *t* test; *, *p* < 0.05; **, *p* < 0.01. The increase in phosphorylation was first detected in olfactory bulb and frontal cortex, followed by the temporal and occipital cortices. Signal intensities of pSer46-MARCKS increased rapidly in the temporal and occipital regions and became most prominent among multiple brain regions. (See also Figs. 3-1, 3-2, and 3-3.)

To determine the initial time point of the increase of pSer46-MARCKS in the brain, we analyzed human α-Syn-BAC-Tg/GBA-hetero-KO mice at 1, 6, and 24 months of age, when the animals did not exhibit motor dysfunction or behavioral abnormality. The mice had slightly elevated signals of pSer46-MARCKS in the olfactory bulb, frontal cortex, and parietal cortex at 1 month (Figs. 3 and 3-1). Subsequently, the area of pSer46-MARCKS–positive staining expanded to parietal and occipital cortex at 6 months (Figs. 3 and 3-2), but did not increase in the hippocampus before 24 months of age (Figs. 3 and 3-3).

10.1523/ENEURO.0217-18.2018.f3-1 Figure 3-1 pSer46-MARCKS in human α-Syn-BAC-Tg/GBA-hetero-KO mice at 1 month of age. pSer46-MARCKS and pSer129-α-Syn were costained in human normal α-Syn-BAC-Tg/glucocerebrosidase (GBA)-hetero-KO mice at 1 month of age (3 males in each group). Signal intensities were significantly higher in yellow-marked areas. Download Figure 3-1, TIF file.

10.1523/ENEURO.0217-18.2018.f3-2 Figure 3-2 pSer46-MARCKS in human α-Syn-BAC-Tg/GBA-hetero-KO mice at 6 months of age. pSer46-MARCKS and pSer129-α-Syn were costained in human normal α-Syn-BAC-Tg/glucocerebrosidase (GBA)-hetero-KO mice at 6 months of age (3 males in each group). Signal intensities were significantly higher in yellow-marked areas. Download Figure 3-2, TIF file.

10.1523/ENEURO.0217-18.2018.f3-3 Figure 3-3 pSer46-MARCKS in human α-Syn-BAC-Tg/GBA-hetero-KO mice at 24 months of age. pSer46-MARCKS and pSer129-α-Syn were costained in human normal α-Syn-BAC-Tg/glucocerebrosidase (GBA)-hetero-KO mice at 24 months of age (3 males in each group). Signal intensities were significantly higher in yellow-marked areas. Download Figure 3-3, TIF file.

**Figure 4.**
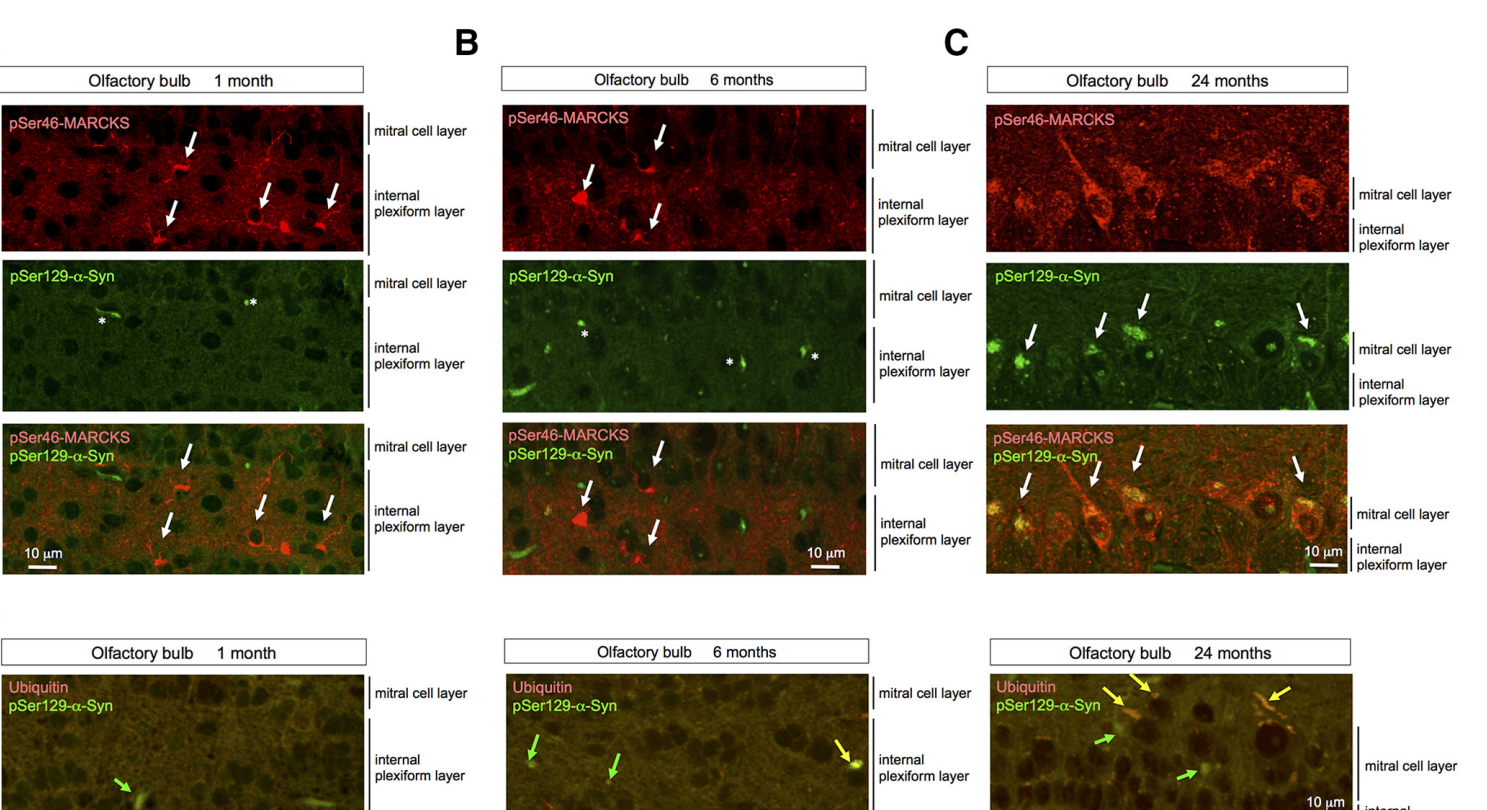
Costaining of pSer46-MARCKS and pSer129-α-Syn in olfactory bulb of human α-Syn-BAC-Tg/GBA-hetero-KO mice. ***A–C***, High magnification of olfactory bulb of human α-Syn-BAC-Tg/GBA-hetero-KO mice costained with antibodies against pSer46-MARCKS and pSer129-α-Syn. Cytoplasmic staining of pSer46-MARCKS (white arrow) and dot-like stains of pSer129-α-Syn (asterisk) were detected from 1 month of age, whereas cytoplasmic aggregates of pSer129-α-Syn were detected only at 24 months. ***D***, Ubiquitin was costained as dot-like structures or cytoplasmic aggregates in a subset of cells (yellow arrow), whereas pSer129-α-Syn–positive/ubiquitin-negative dots or aggregates were also observed.

No definite cytoplasmic α-Syn aggregates stained by anti-phosphorylated α-Syn (pSer129-α-Syn) antibody were detected in neurons of the internal plexiform layer and mitral cell layer of the olfactory bulb until 24 months of age (Fig. 4*A–C*), whereas α-Syn-skein– or Lewy neurite–like structures were present in these layers from 1 month of age, and increased in abundance over the course of aging. These structures were not costained with anti-ubiquitin antibody at 1 month, but costaining was evident from 6 months onward (Fig. 4*D*). Interestingly, pSer46-MARCKS–positive neurites were more frequent than *p*-α-Syn–costained neurites. Together, these results indicated that neurite changes detected by pSer46-MARCKS preceded formation of ubiquitin-positive aggregates of α-Syn, at least in this PD/DLB mouse model (Table 1).

**Table 1.**
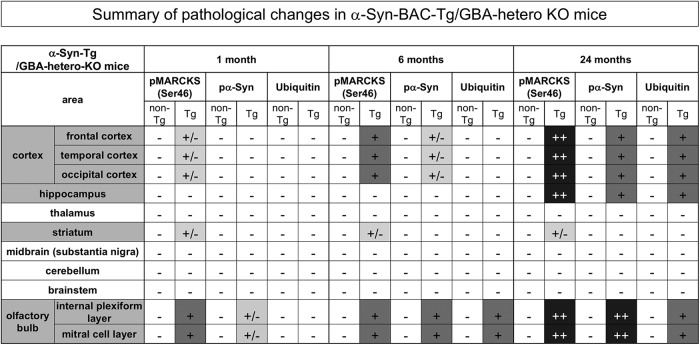
Summary of pSer46-MARCKS, pSer129-α-syn, and ubiquitin staining patterns in human α-syn-BAC-Tg/GBA-hetero-KO mice.

±, positive staining not observed in background mice was observed in <10% of cells; +, positive staining was observed in 10%–50% of cells of Tg mice; ++, positive staining was observed in >50% of cells of Tg mice.

**Figure 5.**
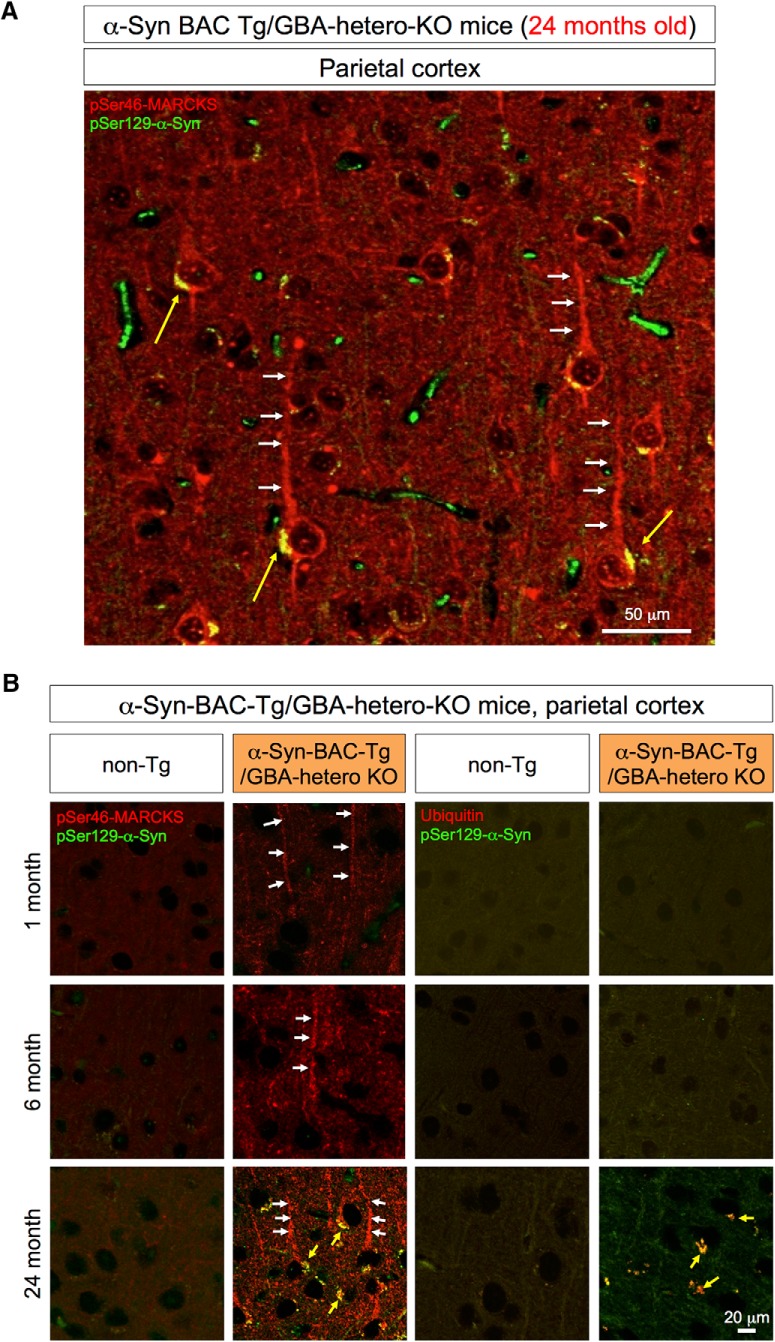
Immunohistochemistry of the parietal lobe of α-Syn-BAC-Tg/GBA-hetero-KO mice. ***A***, pSer46-MARCKS and pSer129-α-Syn were costained in external and internal pyramidal cell layers at 24 months of age. pSer46-MARCKS was stained in both apical dendrites and cell bodies, whereas pSer129-α-Syn was stained in cytoplasmic aggregates. ***B***, Parietal cortex tissues from human α-Syn-BAC-Tg/GBA-hetero-KO mice were costained for pSer129-α-Syn and pSer46-MARCKS or ubiquitin at 1, 6, and 24 months of age. Costaining patterns similar to those in the olfactory bulb were confirmed in the parietal lobe.

At 24 months of age, cytoplasmic α-Syn aggregates were also detected in parietal cortex (Fig. 5*A*). The relationships between pMARCKS and *p*-α-Syn and between *p*-α-Syn and ubiquitin in the cortical neurons during aging were similar to those in olfactory neurons (Fig. 5*B*).

**Figure 6.**
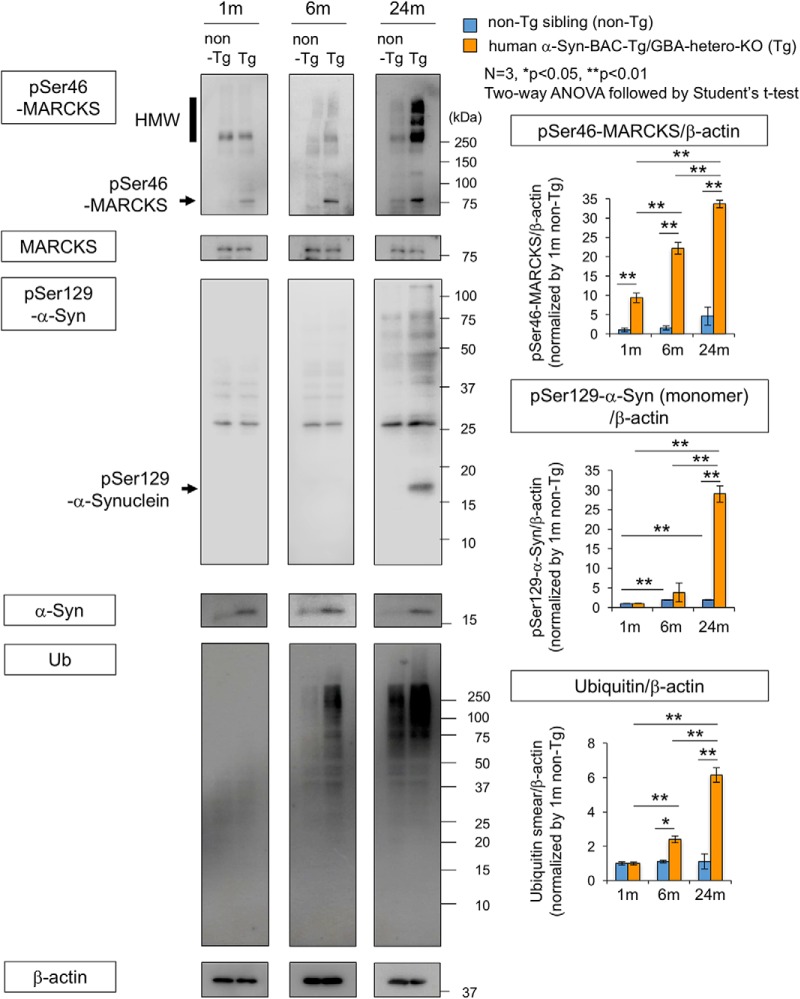
Western blot analysis of α-Syn-BAC-Tg/GBA-hetero-KO mice at multiple time points. Left panels show Western blots of whole cortex (3 males at each time point) with antibodies against pSer46-MARCKS, pSer129-α-Syn, and ubiquitin. Right graphs show quantitative analyses of three independent blots for pSer46-MARCKS, pSer129-α-Syn, and ubiquitin; band intensities were normalized against β-actin. Statistical analyses were performed with two-way ANOVA followed by Student’s *t* test; *, *p* < 0.05; **, *p* < 0.01. (See also Fig. 6-1.)

To confirm these results, we performed Western blot analysis of pSer46-MARCKS, pSer129-α-Syn, and ubiquitin with total cerebral cortex tissues at 1, 6, and 24 months of age (Fig. 6). The level of phosphorylated MARCKS was elevated at 1, 6, and 24 months of age, whereas phosphorylated α-Syn and ubiquitin arose and increased in abundance after 6 months of age (Fig. 6). We also tested the level of pSer46-MARCKS in peripheral blood cells and found it far lower than that in the brain (Fig. 6-1).

10.1523/ENEURO.0217-18.2018.f6-1 Figure 6-1 Protein levels of pSer46-MARCKS were compared between peripheral blood cells (PBC) and whole cerebral cortex (brain) of α-Syn-BAC-Tg/GBA-hetero-KO (Tg) or the nontransgenic sibling control (non-Tg) mice at 6 months of age. Download Figure 6-1, TIF file.

### Interaction between MARCKS and α-Syn in DLB brains

**Figure 7.**
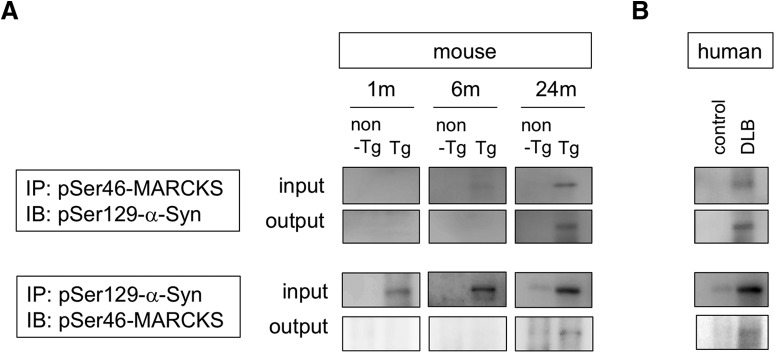
Interaction between MARCKS and α-Syn. ***A***, Immunoprecipitates by anti-pSer46-MARCKS or anti-pSer129-α-Syn antibody from brain samples of α-Syn-BAC-Tg/GBA-hetero-KO mice at 1, 6, and 24 months were blotted with anti-pSer129-α-Syn or anti-pSer46-MARCKS antibody, respectively. ***B***, The similar coprecipitations from temporal lobe cortexes of human DLB patients were examined.

Given that half of the cytoplasmic aggregates were double positive for pSer46-MARCKS and pSer129-α-Syn (Fig. 2*D*), we examined biochemical interaction of the two phosphoproteins. Immunoprecipitation by anti–pSer46-MARCKS coprecipitated pSer129-α-Syn from brain samples (whole cerebral cortex) of α-Syn-BAC-Tg/GBA-hetero-KO mice at 24 months of age (Fig. 7*A*) and from those of human DLB patients (Fig. 7*B*). The reverse precipitation by anti–pSer129-α-Syn antibody also coprecipitated pSer46-MARCKS in both the mouse model and human patients (Fig. 7*A*, *B*), indicating biochemical interaction of the two phosphoproteins.

### Activation of the upstream kinases in DLB brains

Finally, we investigated the upstream kinases that phosphorylate MARCKS at Ser46. MARCKS is a representative substrate of PKC (Aderem, 1992; Blackshear, 1993), as known from its name, myristoylated alanine-rich C kinase substrate. It was reported that PKCα phosphorylates Ser159, Ser163, and Ser170 (Calabrese and Halpain, 2005). Meanwhile, our previous experiments revealed that Erk1/Erk2 (MAPK3/MAPK1) instead of PKC phosphorylates MARCKS at Ser46 (Fujita et al., 2016). Consistently, another group also showed that MAPK phosphorylates MARCKS in hippocampal neurons, although the exact phosphorylation sites by MAPK were determined in their study (Ohmitsu et al., 1999).

**Figure 8.**
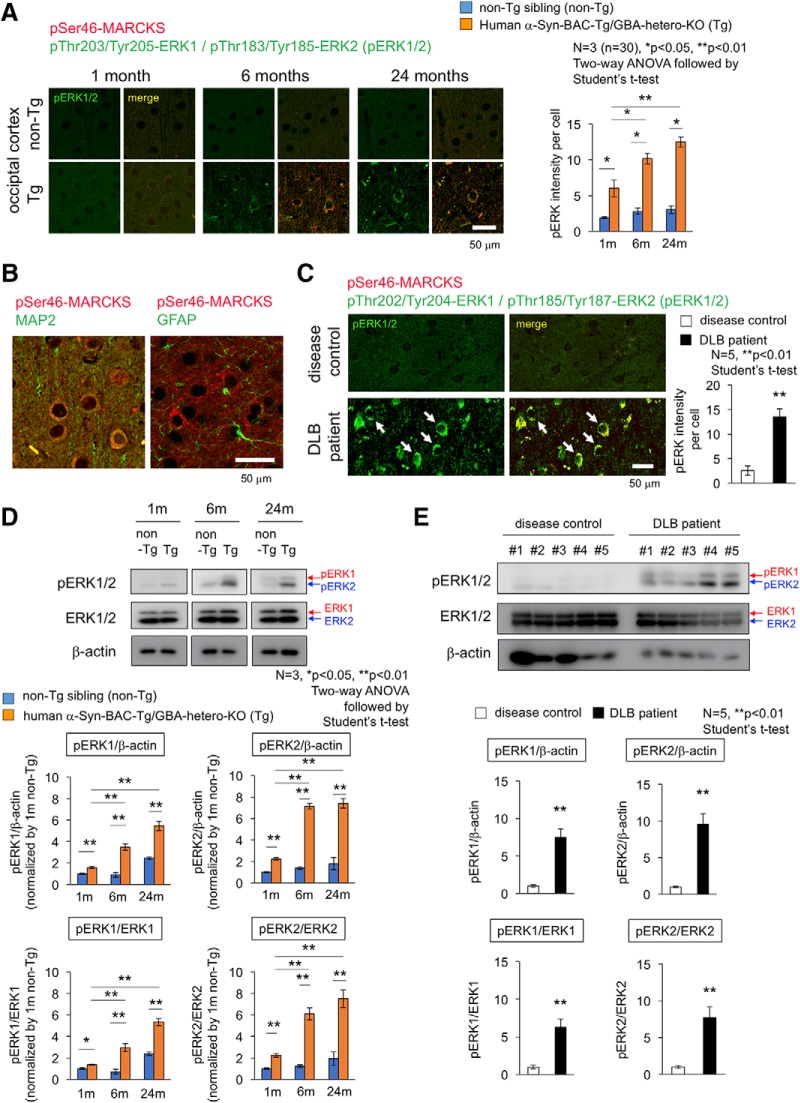
Activation of Erk1/2 in cortical neurons of mouse PD/DLB model and human DLB patient. ***A***, Costaining of occipital cortex tissues from α-Syn-BAC-Tg/GBA-hetero-KO (Tg) or the nontransgenic sibling control (non-Tg) mice at 1, 6, and 24 months of age, by antibodies against Erk1/2 active forms (pThr202/Tyr204-Erk1 and pThr185/Tyr187-Erk2) and against pSer46-MARCKS. Right graph shows quantitative analysis of pErk1/2 signal intensities in 3 mice (10 visual fields for mean value of each mouse). Statistical analyses were performed with two-way ANOVA followed by Student’s *t* test; *, *p* < 0.05; **, *p* < 0.01. ***B***, Costaining of pSer46-MARCKS with MAP2 or GFAP in mouse cortex. ***C***, The similar costaining of pErk1/2 and pSer46-MARCKS in human temporal lobe from DLB patients and non-neurologic disease controls. ***D***, Western blot analyses of whole cortex tissues from α-Syn-BAC-Tg/GBA-hetero-KO (Tg) or non-Tg mice at 1, 6, and 24 months of age with anti-pErk1/2 and -Erk1/2 antibodies. ***E***, The similar Western blot analyses of temporal lobes from human DLB patients.

Therefore, we examined activation of Erk1/2 by immunohistochemistry and Western blot (Fig. 8) by using a rabbit monoclonal antibody detecting Erk1 phosphorylation at Thr202/Tyr204 and Erk2 phosphorylation at Thr185/Tyr187. As expected, immunostaining of pErk1/2 was increased in cerebral cortex of α-Syn-BAC-Tg/GBA-hetero-KO mice (Fig. 8*A*). The cells were costained with MAP2 but not with GFAP and were shown to be neurons (Fig. 8*B*). The stains of pErk1/2 were colocalized with pSer46-MARCKS in the same neurons (Fig. 8*A*), consistent with their enzyme-substrate relationship. The similar costains of increased pSer46-MARCKS and pErk1/2 were confirmed in temporal lobe cortex of DLB patients (Fig. 8*C*). Western blot analyses also confirmed the increase of pErk1/2 in the cerebral cortex of α-Syn-BAC-Tg/GBA-hetero-KO mice (Fig. 8*D*) and DLB patients (Fig. 8*E*). These analyses collectively revealed abnormal increase of Erk1/2 phosphorylation and its age-dependent enhancement in cortical neuron under the PD/DLB pathology (Fig. 8).

## Discussion

In this study, we revealed that the level of MARCKS phosphorylated at pSer46, a hallmark of neurite degeneration at the pre-aggregation stage of AD pathology (Fujita et al., 2016), was also elevated in PD/DLB pathologies in both mouse models and human patients. The immunohistochemical staining patterns of pSer46-MARCKS were also similar in AD and PD/DLB pathologies. In a mouse model, neurite degeneration revealed by pSer46-MARCKS was initially detected in the olfactory bulb and subsequently became prominent in the occipital and temporal cortices (Table 1). This pattern was consistent with the proposed progression of neurodegeneration in human PD/DLB pathology, in which the earliest lesions appear in the olfactory bulb, and the occipital lobe is ultimately predominant (Barber et al., 2000; Lobotesis et al., 2001; Burton et al., 2002, [Bibr B8][Bibr B9][Bibr B10]; Cousins et al., 2003; Ballmaier et al., 2004; Hanyu et al., 2005; Barkhof et al., 2007; Beyer et al., 2007; Whitwell et al., 2007; Perneczky et al., 2008; Klein et al., 2010; Lee et al., 2010; Sanchez-Castaneda et al., 2010; Galvin et al., 2011; Chow et al., 2012; Hayashi et al., 2012; Kantarci et al., 2012; Watson and O’Brien, 2012; Lebedev et al., 2013).

The second critical conclusion of this study is that neurite degeneration precedes disease-related protein aggregation at the histologic level. In the mouse model, the increase in pSer46-MARCKS preceded formation of ubiquitinated α-Syn aggregates (Table 1). The chronological order in the PD/DLB mouse model, in which pSer46-MARCKS precedes histologic aggregate formation, was analogous to our previous observation in AD model mice, in which pSer46-MARCKS was detected in the cytoplasm and neurites of neurons before staining of extracellular Aβ aggregates was apparent (Fujita et al., 2016). Using this model, we confirmed the importance of pSer46-MARCKS at the biochemical level. These results strongly suggest that intracellular misfolded α-Syn in the monomeric or oligomeric form, rather than aggregates, plays the more critical role in initiation of neurite degeneration.

Interestingly, in human postmortem brains, pSer46-MARCKS was elevated in brain regions relatively unaffected by the disease (temporal lobe in DLB, occipital lobe in AD) rather than in severely affected regions (occipital lobe in DLB, temporal lobe in AD). The reason for this has not yet been determined. One possibility is that neurites in severely affected brain regions are metabolically “burned out” and cannot maintain high levels of pSer46-MARCKS.

Based on these observations, it would be worthwhile to develop pSer46-MARCKS as a biomarker capable of detecting the molecular pathology in PD/DLB at the ultra-early (pre-aggregation/preclinical) phase. In addition, it would be useful to develop mass spectrometry–based and ELISA-based assays and/or positron emission tomography. If such a highly sensitive assay system were to become available, it would lead directly to detection of ultra-early-phase pathology in people at risk of neurodegenerative diseases. Preliminarily, we examined by Western blot whether peripheral blood cells (PBCs) such as red blood cells, granule cells, or lymphocytes express pSer46-MARCKS. If it is the case, discrimination of brain-derived from PBC-derived pSer46-MARCKS becomes difficult, and we need some additional tricks to use pSer46-MARCKS as a diagnostic biomarker outside of the brain. Fortunately PBCs did not express a detectable level of pSer46-MARCKS comparable to brain tissues of 5xFAD mice (Fig. 6-1), supporting the possibility to develop pSer46-MARCKS as a blood biomarker.

We found colocalization in cytoplasmic aggregates (Figs. 4 and 5) and biochemical interaction (Fig. 7) of pSer46-MARCKS and pSer129-α-Syn. Interestingly, both α-Syn and MARCKS seem to be intrinsically disordered proteins (IDPs). Previous investigations revealed that α-Syn has a naturally denaturing characteristic (Dedmon et al., 2005; Li et al., 2008; Waudby et al., 2013; Deckert et al., 2016). Meanwhile, bioinformatics analysis with available algorithms (RONN v3.2, https://www.strubi.ox.ac.uk/RONN, and IUPred, http://iupred2a.elte.hu) predicted MARCKS to be IDP (data not shown), and some previous reports supported this idea by structural biology experiments (Arbuzova et al., 2002). The similar denaturing propensity of α-Syn and MARCKS might underlie their biochemical interaction. It is also of note that both α-Syn and MARCKS are localized in degenerative neurites (Masliah et al., 1996; Takeda et al., 1998; Newell et al., 1999; Fujita et al., 2016) and are implicated in axon terminal functions such as axonal growth (Takenouchi et al., 2001; Sousa et al., 2009) and actin network regulation (Sousa et al., 2009; Fujita et al., 2016).

In this study, we focused on pSer46-MARCKS but did not extend our analyses to other molecules detected in the phosphoproteome analysis (Fig. 1-1). Another potentially interesting commonality between AD and PD/DLB is phosphorylation of phosphoglucomutase-1 (PGM1) at Ser117 in the temporal and occipital lobes in patients with both diseases. This enzyme is essential for the conversion of glucose 1-phosphate (generated by glycogenolysis) to glucose 6-phosphate, which is used for energy production by the glycolytic pathway. Further studies are necessary to evaluate the pathologic significance of changes in the phosphorylation-state changes of such additional candidate proteins.

## Synthesis

Reviewing Editor: Jörg Schulz, RWTH Aachen University Decisions are customarily a result of the Reviewing Editor and the peer reviewers coming together and discussing their recommendations until a consensus is reached. When revisions are invited, a fact-based synthesis statement explaining their decision and outlining what is needed to prepare a revision will be listed below. The following reviewer(s) agreed to reveal their identity: NONE.

Protein aggregation is a hallmark of several neurodegenerative diseases including Alzheimer's disease (AD). The phosphorylation of myristoylated alanine-rich C kinase substrate (MARCKS) has been identified as an early indicator of neurodegeneration in AD. In this study, the authors hypothesized that pMARCKS may have the same qualities as a marker in Parkinson's disease or dementia with Lewy body (DLB), which are also characterized by protein aggregates.

The authors carried out a phosphoproteome analysis of control, AD and DLB patients and found that pSer46-MARCKS was increased. To monitor the upregulation of MARCKS phosphorylation, they took advantage of an PD/DLB transgenic mouse model and performed histological analyses of pMARCKS in young, middle-aged and aged mice. Interestingly, pMARCKS was detectable at 1 month of age, a time point when pSer129-α-synuclein has not appeared, yet. In further immunoblotting experiments, they confirmed the upregulation of pMARCKS (and stable total MARCKS levels) in cortical lysates. The authors also showed the appearance of pSer129-α-synuclein at a later time, and the increase in ubiquitin in this mouse model. Finally, the authors validate the upregulation of pSer46-MARCKS in human brain tissue of control and DLB patients both with histological techniques and immunoblotting.

In conclusion, the results of the study support the notion that pSer46MARCKS could serve as a disease marker in PD/DLB.

Comments:

- MARCKS pathway: Is PKC the kinase that mediates the P at Ser46? What about the activity status of PKC in the mouse brain (or human brains)? Is there increased phosphorylation of other PKC substrates?

- Fig #6 serves as the critical validation of the phosphoproteome analysis shown in Fig #1 and should appear earlier.

- Why is there no ubiquitin signal in the control brain lysates shown in Fig #5? . Ubiquitin antibodies typically produce a smear. Whether or not the signal is upregulated depends on the extent of ubiquitin accumulation. The blank lanes of the control groups raise some doubts.

- Would pMARCKS be suitable as diagnostic marker outside the brain? Is it expressed in PBCs?

- Is there a biochemical relationship between MARKCS and alpha-synuclein?

- presentation of quantifications in Fig #2 and #5. The results were analyzed using a Student's T-test. The data however are presented in such a way that the analysis requires a two-way ANOVA (two groups and three time points)!

## References

[B1] Aderem A (1992) The MARCKS brothers: a family of protein kinase C substrates. Cell 71:713–716. 1423627 10.1016/0092-8674(92)90546-o

[B2] Arbuzova A, Schmitz AA, Vergères G (2002) Cross-talk unfolded: MARCKS proteins. Biochem J 362:1–12. 11829734 10.1042/0264-6021:3620001 PMC1222354

[B3] Ballmaier M, O’Brien JT, Burton EJ, Thompson PM, Rex DE, Narr KL, McKeith IG, DeLuca H, Toga AW (2004) Comparing gray matter loss profiles between dementia with Lewy bodies and Alzheimer’s disease using cortical pattern matching: diagnosis and gender effects. Neuroimage 23:325–335. 10.1016/j.neuroimage.2004.04.026 15325380

[B4] Barber R, Ballard C, McKeith IG, Gholkar A, O’Brien JT (2000) MRI volumetric study of dementia with Lewy bodies: a comparison with AD and vascular dementia. Neurology 54:1304–1309. 10746602 10.1212/wnl.54.6.1304

[B5] Barkhof F, Polvikoski TM, van Straaten EC, Kalaria RN, Sulkava R, Aronen HJ, Niinistö L, Rastas S, Oinas M, Scheltens P, Erkinjuntti T (2007) The significance of medial temporal lobe atrophy: a postmortem MRI study in the very old. Neurology 69:1521–1527. 10.1212/01.wnl.0000277459.83543.99 17923614

[B6] Beyer MK, Larsen JP, Aarsland D (2007) Gray matter atrophy in Parkinson disease with dementia and dementia with Lewy bodies. Neurology 69:747–754. 10.1212/01.wnl.0000269666.62598.1c 17709706

[B7] Blackshear PJ (1993) The MARCKS family of cellular protein kinase C substrates. J Biol Chem 268:1501–1504. 8420923

[B11] Burton EJ, Karas G, Paling SM, Barber R, Williams ED, Ballard CG, McKeith IG, Scheltens P, Barkhof F, O’Brien JT (2002) Patterns of cerebral atrophy in dementia with Lewy bodies using voxel-based morphometry. Neuroimage 17:618–630. 10.1006/nimg.2002.1197 12377138

[B8] Burton EJ, McKeith IG, Burn DJ, Williams ED, O’Brien JT (2004) Cerebral atrophy in Parkinson’s disease with and without dementia: a comparison with Alzheimer’s disease, dementia with Lewy bodies and controls. Brain 127:791–800. 10.1093/brain/awh088 14749292

[B9] Burton EJ, McKeith IG, Burn DJ, Firbank MJ, O’Brien JT (2006) Progression of white matter hyperintensities in Alzheimer disease, dementia with lewy bodies, and Parkinson disease dementia: a comparison with normal aging. Am J Geriatr Psychiatry 14:842–849. 10.1097/01.JGP.0000236596.56982.1c 17001024

[B10] Burton EJ, Barber R, Mukaetova-Ladinska EB, Robson J, Perry RH, Jaros E, Kalaria RN, O’Brien JT (2009) Medial temporal lobe atrophy on MRI differentiates Alzheimer’s disease from dementia with Lewy bodies and vascular cognitive impairment: a prospective study with pathological verification of diagnosis. Brain 132:195–203. 10.1093/brain/awn298 19022858

[B12] Calabrese B, Halpain S (2005) Essential role for the PKC target MARCKS in maintaining dendritic spine morphology. Neuron 48:77–90. 10.1016/j.neuron.2005.08.027 16202710

[B13] Chow N, Aarsland D, Honarpisheh H, Beyer MK, Somme JH, Elashoff D, Rongve A, Tysnes OB, Thompson PM, Apostolova LG (2012) Comparing hippocampal atrophy in Alzheimer’s dementia and dementia with lewy bodies. Dement Geriatr Cogn Disord 34:44–50. 10.1159/000339727 22922563 PMC3470878

[B14] Cousins DA, Burton EJ, Burn D, Gholkar A, McKeith IG, O’Brien JT (2003) Atrophy of the putamen in dementia with Lewy bodies but not Alzheimer’s disease: an MRI study. Neurology 61:1191–1195. 14610119 10.1212/01.wnl.0000091889.20347.30

[B15] Deckert A, Waudby CA, Wlodarski T, Wentink AS, Wang X, Kirkpatrick JP, Paton JF, Camilloni C, Kukic P, Dobson CM, Vendruscolo M, Cabrita LD, Christodoulou J (2016) Structural characterization of the interaction of alpha-synuclein nascent chains with the ribosomal surface and trigger factor. Proc Natl Acad Sci U S A 113:5012–5017. 10.1073/pnas.1519124113 27092002 PMC4983817

[B16] Dedmon MM, Lindorff-Larsen K, Christodoulou J, Vendruscolo M, Dobson CM (2005) Mapping long-range interactions in alpha-synuclein using spin-label NMR and ensemble molecular dynamics simulations. J Am Chem Soc 127:476–477. 10.1021/ja044834j 15643843

[B17] Fujita K, Motoki K, Tagawa K, Chen X, Hama H, Nakajima K, Homma H, Tamura T, Watanabe H, Katsuno M, Matsumi C, Kajikawa M, Saito T, Saido T, Sobue G, Miyawaki A, Okazawa H (2016) HMGB1, a pathogenic molecule that induces neurite degeneration via TLR4-MARCKS, is a potential therapeutic target for Alzheimer’s disease. Sci Rep 6:31895. 10.1038/srep31895 27557632 PMC4997258

[B18] Galvin JE, Price JL, Yan Z, Morris JC, Sheline YI (2011) Resting bold fMRI differentiates dementia with Lewy bodies vs Alzheimer disease. Neurology 76:1797–1803. 10.1212/WNL.0b013e31821ccc83 21525427 PMC3100121

[B19] Hanyu H, Tanaka Y, Shimizu S, Sakurai H, Iwamoto T, Abe K (2005) Differences in MR features of the substantia innominata between dementia with Lewy bodies and Alzheimer’s disease. J Neurol 252:482–484. 10.1007/s00415-005-0611-8 15772744

[B20] Hayashi H, Kawakatsu S, Suzuki A, Shibuya Y, Kobayashi R, Sato C, Otani K (2012) Application of the VSRAD, a specific and sensitive voxel-based morphometry, to comparison of entorhinal cortex atrophy between dementia with Lewy bodies and Alzheimer’s disease. Dement Geriatr Cogn Disord 34:328–331. 10.1159/000345792 23208522

[B21] Kantarci K, Ferman TJ, Boeve BF, Weigand SD, Przybelski S, Vemuri P, Murray ME, Senjem ML, Smith GE, Knopman DS, Petersen RC, Jack CR, Jr., Parisi JE, Dickson DW (2012) Focal atrophy on MRI and neuropathologic classification of dementia with Lewy bodies. Neurology 79:553–560. 10.1212/WNL.0b013e31826357a5 22843258 PMC3413765

[B22] Klein JC, Eggers C, Kalbe E, Weisenbach S, Hohmann C, Vollmar S, Baudrexel S, Diederich NJ, Heiss WD, Hilker R (2010) Neurotransmitter changes in dementia with Lewy bodies and Parkinson disease dementia in vivo. Neurology 74:885–892. 10.1212/WNL.0b013e3181d55f61 20181924

[B23] Lebedev AV, Westman E, Beyer MK, Kramberger MG, Aguilar C, Pirtosek Z, Aarsland D (2013) Multivariate classification of patients with Alzheimer’s and dementia with Lewy bodies using high-dimensional cortical thickness measurements: an MRI surface-based morphometric study. J Neurol 260:1104–1115. 10.1007/s00415-012-6768-z 23224109

[B24] Lee JE, Park B, Song SK, Sohn YH, Park HJ, Lee PH (2010) A comparison of gray and white matter density in patients with Parkinson’s disease dementia and dementia with Lewy bodies using voxel-based morphometry. Mov Disord 25:28–34. 10.1002/mds.22858 19908327

[B25] Li C, Charlton LM, Lakkavaram A, Seagle C, Wang G, Young GB, Macdonald JM, Pielak GJ (2008) Differential dynamical effects of macromolecular crowding on an intrinsically disordered protein and a globular protein: implications for in-cell NMR spectroscopy. J Am Chem Soc 130:6310–6311. 10.1021/ja801020z 18419123 PMC2435198

[B26] Lobotesis K, Fenwick JD, Phipps A, Ryman A, Swann A, Ballard C, McKeith IG, O’Brien JT (2001) Occipital hypoperfusion on SPECT in dementia with Lewy bodies but not AD. Neurology 56:643–649. 11245717 10.1212/wnl.56.5.643

[B27] Masliah E, Iwai A, Mallory M, Uéda K, Saitoh T (1996) Altered presynaptic protein NACP is associated with plaque formation and neurodegeneration in Alzheimer’s disease. Am J Pathol 148:201–210. 8546207 PMC1861620

[B28] Newell KL, Boyer P, Gomez-Tortosa E, Hobbs W, Hedley-Whyte ET, Vonsattel JP, Hyman BT (1999) Alpha-synuclein immunoreactivity is present in axonal swellings in neuroaxonal dystrophy and acute traumatic brain injury. J Neuropathol Exp Neurol 58:1263–1268. 10604751 10.1097/00005072-199912000-00007

[B29] Ohmitsu M, Fukunaga K, Yamamoto H, Miyamoto E (1999) Phosphorylation of myristoylated alanine-rich protein kinase C substrate by mitogen-activated protein kinase in cultured rat hippocampal neurons following stimulation of glutamate receptors. J Biol Chem 274:408–417. 9867858 10.1074/jbc.274.1.408

[B30] Perneczky R, Drzezga A, Boecker H, Förstl H, Kurz A, Häussermann P (2008) Cerebral metabolic dysfunction in patients with dementia with Lewy bodies and visual hallucinations. Dement Geriatr Cogn Disord 25:531–538. 10.1159/000132084 18477846

[B31] Sanchez-Castaneda C, Rene R, Ramirez-Ruiz B, Campdelacreu J, Gascon J, Falcon C, Calopa M, Jauma S, Juncadella M, Junque C (2010) Frontal and associative visual areas related to visual hallucinations in dementia with Lewy bodies and Parkinson’s disease with dementia. Mov Disord 25:615–622. 10.1002/mds.22873 20175186

[B32] Shilov IV, Seymour SL, Patel AA, Loboda A, Tang WH, Keating SP, Hunter CL, Nuwaysir LM, Schaeffer DA (2007) The Paragon Algorithm, a next generation search engine that uses sequence temperature values and feature probabilities to identify peptides from tandem mass spectra. Mol Cell Proteomics 6:1638–1655. 10.1074/mcp.T600050-MCP200 17533153

[B33] Sousa VL, Bellani S, Giannandrea M, Yousuf M, Valtorta F, Meldolesi J, Chieregatti E (2009) alpha-Synuclein and its A30P mutant affect actin cytoskeletal structure and dynamics. Mol Biol Cell 20:3725–3739. 10.1091/mbc.e08-03-0302 19553474 PMC2777932

[B34] Tagawa K, Homma H, Saito A, Fujita K, Chen X, Imoto S, Oka T, Ito H, Motoki K, Yoshida C, Hatsuta H, Murayama S, Iwatsubo T, Miyano S, Okazawa H (2015) Comprehensive phosphoproteome analysis unravels the core signaling network that initiates the earliest synapse pathology in preclinical Alzheimer’s disease brain. Hum Mol Genet 24:540–558. 10.1093/hmg/ddu475 25231903

[B35] Takeda A, Mallory M, Sundsmo M, Honer W, Hansen L, Masliah E (1998) Abnormal accumulation of NACP/alpha-synuclein in neurodegenerative disorders. Am J Pathol 152:367–372. 9466562 PMC1857971

[B36] Takenouchi T, Hashimoto M, Hsu LJ, Mackowski B, Rockenstein E, Mallory M, Masliah E (2001) Reduced neuritic outgrowth and cell adhesion in neuronal cells transfected with human alpha-synuclein. Mol Cell Neurosci 17:141–150. 10.1006/mcne.2000.0923 11161475

[B37] Watson R, O’Brien JT (2012) Differentiating dementia with Lewy bodies and Alzheimer’s disease using MRI. Neurodegen Dis Manage 2:411–420. 10.2217/nmt.12.41

[B38] Waudby CA, Camilloni C, Fitzpatrick AW, Cabrita LD, Dobson CM, Vendruscolo M, Christodoulou J (2013) In-cell NMR characterization of the secondary structure populations of a disordered conformation of alpha-synuclein within E. coli cells. PLoS One 8:e72286. 10.1371/journal.pone.0072286 23991082 PMC3753296

[B39] Whitwell JL, Weigand SD, Shiung MM, Boeve BF, Ferman TJ, Smith GE, Knopman DS, Petersen RC, Benarroch EE, Josephs KA, Jack CR, Jr. (2007) Focal atrophy in dementia with Lewy bodies on MRI: a distinct pattern from Alzheimer’s disease. Brain 130:708–719. 10.1093/brain/awl388 17267521 PMC2730778

[B40] Yamakado H, Moriwaki Y, Yamasaki N, Miyakawa T, Kurisu J, Uemura K, Inoue H, Takahashi M, Takahashi R (2012) alpha-Synuclein BAC transgenic mice as a model for Parkinson’s disease manifested decreased anxiety-like behavior and hyperlocomotion. Neurosci Res 73:173–177. 10.1016/j.neures.2012.03.010 22475625

