# Supplementary material for: Ser46-Phosphorylated MARCKS Is a Marker of Neurite Degeneration at the Pre-aggregation Stage in PD/DLB Pathology
Source: eNeuro. 2018 Sep 4;5(4):ENEURO.0217-18.2018. doi: 10.1523/ENEURO.0217-18.2018 (PMC6140116; doi:10.1523/ENEURO.0217-18.2018)
Supplement: Figure 1-1 — Detailed list of phosphorylated proteins in human AD and DLB cortex Download Figure 1-1, DOCX file. [file sup_enu-eN-NWR-0217-18-s01.docx]

**Extended table 1-1. Detailed list of phosphorylated proteins in human AD and DLB cortex**

**DLB** **(occipital** U **temporal)** n **AD** **(occipital** U **temporal)**

| Human AD | | | | | | |
| --- | --- | --- | --- | --- | --- | --- |
| Occipital | | | |  | Temporal | |
| # of p-value peptide Mean (Welch's fragments Ratio test) | | | q-value | # of p-value peptide Mean (Welch's fragments Ratio test) | | q-value |
| 8  39  10  11  8  9  12  82  38  8  18  43  38  57  11  20  11  129  24  136  86  15  41  41  26  252  42  23  12  60  15  24  635  75  423  178  98  53  75  202  202  114  31  687  150  33  5  9  17  55  13  67  41  11  5  8  8 | 6.176634 | 0.001361 0.010352 | |  | | |
|  | 1.874248 | 2.37E-08 7.03E-07 | |  |  |  |
|  | 1.867881 | 0.000252 0.002789 | |  |  |  |
|  | 1.692198 | 0.002724 0.017435 | |  |  |  |
|  | 1.68631 | 0.000314 0.003302 | |  |  |  |
|  | 1.683869 | 1.49E-05 0.000221 | |  |  |  |
|  | 1.642184 | 0.000841 0.007534 | |  |  |  |
|  | 1.587358 | 5.23E-09 1.77E-07 | |  |  |  |
|  | 1.57248 | 2.84E-14 1.69E-12 | |  |  |  |
|  | 1.546928 | 0.003999 0.023745 | |  |  |  |
|  | 1.494913 | 0.002265 0.015151 | |  |  |  |
|  | 1.481222 | 8.79E-06 0.000139 | |  |  |  |
|  | 1.47613 | 4.96E-14 2.62E-12 | |  |  |  |
|  | 1.472853 | 5.79E-13 2.50E-11 | |  |  |  |
|  | 1.428466 | 0.002586 0.016824 | |  |  |  |
|  | 1.416782 | 0.000344 0.003475 | |  |  |  |
|  | 1.405363 | 9.44E-05 0.001246 | |  |  |  |
|  | 1.404552 | 1.64E-18 1.11E-16 | |  |  |  |
|  | 1.377204 | 3.88E-06 6.82E-05 | |  |  |  |
|  | 1.362348 | 3.84E-24 9.12E-22 | |  |  |  |
|  | 1.321039 | 9.62E-08 2.69E-06 | |  |  |  |
|  | 1.311061 | 0.005542 0.028614 | |  |  |  |
|  | 1.30441 | 5.14E-05 0.000718 | |  |  |  |
|  | 1.29062 | 6.95E-05 0.000944 | |  |  |  |
|  | 1.282506 | 0.006402 0.03201 | |  |  |  |
|  | 1.279199 | 2.15E-25 1.02E-22 | |  |  |  |
|  | 1.279116 | 0.004166 0.024132 | |  |  |  |
|  | 1.273167 | 0.001098 0.008714 | |  |  |  |
|  | 1.264465 | 0.000806 0.007425 | |  |  |  |
|  | 1.262485 | 1.03E-06 2.23E-05 | |  |  |  |
|  | 1.259015 | 0.000927 0.007989 | |  |  |  |
|  | 1.247531 | 3.32E-05 0.000478 | |  |  |  |
|  | 1.221528 | 1.76E-23 2.09E-21 | |  |  |  |
|  | 1.212645 | 0.002753 0.017435 | |  |  |  |
|  | 1.201824 | 3.23E-20 3.01E-18 | |  |  |  |
|  | 1.201262 | 7.05E-24 1.12E-21 | |  |  |  |
|  | 1.192191 | 1.24E-05 0.00019 | |  |  |  |
|  | 1.168345 | 0.004329 0.024777 | |  |  |  |
|  | 1.166163 | 0.000202 0.00229 | |  |  |  |
|  | 1.155269  1.155269 | 4.73E-10 1.73E-08  4.73E-10 1.73E-08 | |  |  |  |
|  | 1.130835 | 0.000813 0.007425 | |  |  |  |
|  | 1.123324 | 0.000465 0.004602 | |  |  |  |
|  | 1.097709 | 1.95E-07 4.64E-06 | |  |  |  |
|  | 0.886565 | 0.007147 0.034168 | |  |  |  |
|  | 0.729723 | 0.003257 0.019994 | |  |  |  |
|  | 0.699646 | 0.006556 0.032318 | |  |  |  |
|  | 0.683572 | 0.001485 0.011025 | |  |  |  |
|  | 0.669192 | 0.000107 0.001271 | |  |  |  |
|  | 0.656998 | 1.10E-07 2.91E-06 | |  |  |  |
|  | 0.593443 | 0.004639 0.025325 | |  |  |  |
|  | 0.575271 | 5.64E-07 1.28E-05 | |  |  |  |
|  | 0.543245 | 1.00E-13 4.77E-12 | |  |  |  |
|  | 0.510048 | 0.007654 0.035998 | |  |  |  |
|  | 0.465483 | 0.00477 0.025747 | |  |  |  |
|  | 0.41077 | 0.011642 0.04982 | |  |  |  |
|  | 0.233561 | 0.000942 0.007989 | |  |  |  |

0 NA NA NA

| 0.756899 | 0.003416 0.01297 |
| --- | --- |
| 1.331706 | 3.96E-05 0.000288 |
| 1.396955 | 0.001984 0.008426 |
| 1.20093 | 0.002641 0.01049 |
| 0.562106 | 0.000956 0.004653 |

89

P16949 STMN1 Stathmin S25 11 1.117224 0.424918 0.67818 34

|  | Human DLB | | | | | | |
| --- | --- | --- | --- | --- | --- | --- | --- |
|  | Occipital | | | |  | Temporal | |
| Phosphor # of p-value  UniProtID Gene Full name ylation peptide Mean (Welch's name site fragments Ratio test) | | | | q-value | # of p-value peptide Mean (Welch's fragments Ratio test) | | q-value |
| P60201 PLP1 Myelin pro T193 8  P17600 SYN1 Synapsin- S39 41 | | 4.310976 | 0.000132 0.003191 | |  | | |
|  |  | 1.718327 | 0.000331 0.006938 | |  |  |  |

P46821 MAP1B Microtubul S1262 12 1.335355 0.114814 0.338179 10

O60641 SNAP91 Clathrin coT312 8 0.899786 0.648068 0.83521 25

Q13554 CAMK2B Calcium/c S367 9 1.385075 0.031404 0.156792 4

Q92932 PTPRN2 Receptor-t S360 11 1.488613 0.000945 0.014237 1 0.73652 NA NA P46821 MAP1B Microtubul S1400 84 1.3198 1.77E-06 6.56E-05 86 1.38577 1.08E-08 1.71E-07

P80723 BASP1 Brain acid T196 38 0.739217 0.05021 0.206849 43 0.724446 0.000461 0.002609

P78559 MAP1A Microtubul S1264 8 1.811004 0.000679 0.011548 6 0.874332 0.491905 0.582948

| 0.389673 | 0.016923 0.045375 |
| --- | --- |
| 0.579776 | 0.000647 0.003304 |
| 1.236871 | 0.015744 0.042748 |
| 1.125161 | 0.010134 0.030833 |
| 1.675548 | 1.15E-05 9.85E-05 |
| 1.274974 | 4.66E-05 0.000328 |
| 1.203113 | 0.005718 0.019623 |
| 1.19363 | 5.24E-09 9.53E-08 |

P07197 NEFM NeurofilamS783 19 1.477364 0.023844 0.124924 4

P29966 MARCKS Myristoyla T150 39 0.73679 0.050173 0.206849 40

Q01082 SPTBN1 Spectrin b S2102 38 1.296379 0.003368 0.03959 22

P35612 ADD2 Beta-addu S592 57 1.296665 0.005886 0.054556 73

Q02952 AKAP12 A-kinase a S283 10 1.406958 0.067111 0.246 21

P04406 GAPDH GlyceraldeS210 20 1.365244 0.017146 0.099959 28

P35612 ADD2 Beta-addu S617 11 1.130149 0.534893 0.759677 13

| 1.328256 | 9.04E-08 6.22E-06 |
| --- | --- |
| 1.278518 | 0.002381 0.030619 |
| 1.283901 | 3.07E-07 1.64E-05 |

0 NA NA NA

81 0.709168 0.003574 0.020945

54 0.955496 0.417019 0.596937

10 1.207657 0.054022 0.140131

24 1.154663 0.013935 0.05091

4 0.793376 0.029699 0.089511

1 0.804446 NA NA

89 1.052629 0.191455 0.354731

45 1.072558 0.48504 0.650946

5 0.737867 0.001848 0.012964

5 0.865555 0.518173 0.682258

54 1.393856 0.000189 0.002127

21 0.994303 0.920738 0.945026

67 0.957561 0.245595 0.417394

21 1.282597 0.002364 0.016064

27 1.187302 2.17E-05 0.000321

13 0.854206 0.245755 0.417394

P35611 ADD1 Alpha-add S358 131

Q9Y2J2 EPB41L3 Band 4.1-l S7 27

Q7Z6L0 PRRT2 Proline-ric S208 131

177

9 0.785188 0.079952 0.145336

194 1.164047 1.23E-05 0.000103

168 1.009812 0.732928 0.848849

9 1.229605 0.036357 0.103052

184 0.929378 0.030073 0.089511

P29966 MARCKS Myristoyla S46 88 1.113989 0.100387 0.335407 180 1.211224 1.92E-07 2.35E-06

P17677 GAP43 Neuromod T107 15 0.773262 0.173566 0.422518 9 0.48711 0.000525 0.002813

P51608 MECP2 Methyl-Cp S80 42 1.256945 0.023157 0.124019 22 1.264193 0.019462 0.049996

P09972 ALDOC Fructose-bS39 41 1.270525 0.003625 0.040631 32 1.220351 0.002545 0.010302

P46821 MAP1B Microtubul S995 26 1.107378 0.310613 0.580293 17 0.618827 0.005833 0.01969

P36871 PGM1 Phosphogl S117 256 1.329673 8.82E-13 1.06E-10 153 1.133294 5.33E-09 9.53E-08

P78559 MAP1A Microtubul S2022 43 0.840707 0.010394 0.077076 31 1.275728 0.000968 0.004653

P10636 MAPT Microtubul T498 23 1.132584 0.321506 0.583466 47 1.406638 2.89E-07 3.31E-06

Q9H4G0 EPB41L1 Band 4.1-l S678 14 1.318598 0.054454 0.215666 7 0.560815 0.013491 0.037581

P49418 AMPH Amphiphy S549 60 1.376215 3.20E-06 9.63E-05 19 0.702035 0.000246 0.001488

P07900 HSP90AA Heat shoc S231 15 1.031293 0.772366 0.901405 5 0.528797 0.001059 0.00494

P17252 PRKCA Protein kin S226 26 1.38962 0.000639 0.011405 7 1.131816 0.04863 0.100785

P07197 NEFM NeurofilamS837 686 1.304289 4.97E-24 2.40E-21 91 0.944632 0.05858 0.114268

Q01082 SPTBN1 Spectrin b S2341 75 0.664432 5.56E-07 2.23E-05 35 0.826999 0.036719 0.080369

P16949 STMN1 Stathmin S38 418 0.897392 1.89E-05 0.000536 444 0.869797 0.003554 0.013376

Q8N111 CEND1 Cell cycle S87 178 1.093238 0.006296 0.057255 68 0.849125 1.63E-07 2.05E-06

O94811 TPPP Tubulin po S32 100 0.828094 0.000267 0.005846 20 0.933825 0.407803 0.504171

| 1.261213 | 2.93E-07 3.31E-06 |
| --- | --- |
| 0.877086 | 0.002489 0.010171 |
| 0.914083  0.913881 | 0.000182 0.001119  8.11E-05 0.000561 |

Q9C040 TRIM2 Tripartite S428 54 1.112318 0.143272 0.380557 85

P46821 MAP1B Microtubul S1312 77 1.141216 0.017213 0.099959 33

P63104 YWHAZ 14-3-3 pro S184 204 1.174859 3.94E-07 1.73E-05 124

P31946 YWHAB 14-3-3 pro S186 204 1.174859 3.94E-07 1.73E-05 134

Q16555 DPYSL2 Dihydropy T509 111 0.748261 2.60E-06 8.77E-05 319 0.983617 0.517218 0.602952

| 0.874255 | 0.00247 0.010171 |
| --- | --- |
| 1.360958 | 1.01E-21 4.83E-20 |
| 2.082539 | 1.62E-58 6.96E-56 |
| 1.126276 | 0.000625 0.003272 |
| 1.33462 | 0.002737 0.010674 |

P35611 ADD1 Alpha-add S600 30 0.917686 0.103058 0.335407 16

P07197 NEFM NeurofilamS736 690 1.064583 0.019411 0.106318 177

O00499 BIN1 Myc box-d S298 166 0.929065 0.056464 0.221264 334

P17677 GAP43 Neuromod T181 37 0.635575 0.015843 0.096665 154

Q6H8Q1 ABLIM2 Actin-bindi S294 6 0.779837 0.276154 0.545328 20

P46821 MAP1B Microtubul S1965 10 0.705254 0.004811 0.049684 3 0.832961 0.395531 0.494703

Q16623 STX1A Syntaxin-1 S14 17 0.78383 0.029493 0.15123 15 0.524775 9.16E-07 9.58E-06

Q9UN36 NDRG2 Protein NDS332 64 0.802634 0.005172 0.051021 208 1.679942 1.36E-22 8.36E-21

P46821 MAP1B Microtubul S1817 13 0.585731 0.000415 0.008337 2 0.578185 NA NA P14136 GFAP Glial fibrillaS13 71 0.778043 4.66E-05 0.001247 341

| 1.246914 | 5.18E-17 2.02E-15 |
| --- | --- |
| 0.493081 | 0.013443 0.037581 |
| 1.901219 | 1.70E-11 3.85E-10 |

P23528 CFL1 Cofilin-1 S3 40 0.568514 0.002212 0.02961 6

P29966 MARCKS Myristoyla S118 12 0.659674 0.101099 0.335407 60

Q13263 TRIM28 Transcripti S19 5 0.35411 0.004009 0.043914 0 NA NA NA P10636 MAPT Microtubul S531 8 0.956259 0.876781 0.943322 56 1.425878 5.35E-06 5.22E-05

P11137 MAP2 Microtubul S833 8 0.376407 0.013865 0.089103 49 0.771519 0.018837 0.048976

170 0.934439 0.007254 0.033029

10 0.783972 0.082861 0.187643

23 1.273084 0.001118 0.00903

31 1.039981 0.440713 0.618089

16 0.770824 2.95E-05 0.000421

151 1.09733 0.000152 0.001865

31 1.116821 0.06443 0.160326

47 0.911696 0.311499 0.493065

7 0.900087 0.514148 0.681286

19 0.716117 3.62E-06 7.75E-05

5 0.502082 0.004645 0.024165

7 1.018045 0.869323 0.923251

97 1.162767 2.32E-06 5.24E-05

35 1.110769 0.180833 0.343984

436 1.098652 1.28E-13 8.61E-12

68 0.898863 0.077321 0.178882

18 0.935105 0.469599 0.638059

80 1.152987 0.000457 0.004547

28 0.783697 0.003314 0.020265

121 0.764323 7.14E-13 3.82E-11

131 0.770674 1.41E-13 8.61E-12

305 1.053995 0.022131 0.074002

15 0.741119 0.001212 0.009386

178 1.534712 1.10E-21 1.57E-19

320 1.466685 9.11E-25 3.90E-22

138 1.034417 0.452286 0.626476

16 1.14271 0.139089 0.280803

3 0.832354 0.237143 0.409264

15 0.518578 2.87E-08 8.80E-07

175 1.185832 5.89E-05 0.000743

2 0.857774 NA NA

313 1.176298 6.88E-08 1.84E-06

6 0.964146 0.773234 0.861886

56 0.762605 3.48E-08 9.94E-07

0 NA NA NA

52 0.843417 0.363933 0.548463

50 0.895691 0.434968 0.616444

| 2.014409 | 0.000339 0.00354 |
| --- | --- |
| 1.39923 | 0.000188 0.002127 |
| 1.315897 | 0.00373 0.020994 |
| 1.118111 | 0.00367 0.020945 |
| 1.158294 | 0.007331 0.033029 |
| 1.158294 | 0.007331 0.033029 |
| 1.943769 | 0.003093 0.020058 |
| 1.174193 | 0.009544 0.038599 |
| 1.323532 | 1.86E-05 0.000295 |
| 0.770591 | 0.00366 0.020945 |
| 0.760619 | 0.001228 0.009386 |
| 1.122026 | 1.93E-06 4.60E-05 |
| 1.234383 | 7.27E-06 0.000141 |
| 0.838198 | 0.000224 0.002399 |
| 1.088428 | 0.013134 0.048462 |
| 1.636194 | 0.003295 0.020265 |
| 1.200343 | 0.004743 0.024165 |
| 1.140161 | 0.002262 0.015614 |
| 0.852442 | 6.91E-11 3.29E-09 |
| 0.84548 | 0.00965 0.038599 |
| 1.231366 | 2.88E-08 8.80E-07 |
| 0.152934 | 0.008488 0.035968 |
| 1.148606 | 0.004348 0.023262 |
| 0.593971 | 0.000691 0.006174 |
| 1.286313 | 0.01043 0.040954 |
| 0.740102 | 0.001536 0.010954 |
| 0.266769 | 1.05E-05 0.000195 |
| 1.209975 | 0.000662 0.006163 |
| 0.701072 | 0.011703 0.043939 |
| 1.477753 | 1.37E-10 5.84E-09 |
| 0.685814 | 3.03E-14 3.24E-12 |
| 0.880573 | 4.58E-09 1.78E-07 |
| 0.754464 | 0.008595 0.036066 |
| 0.694455 | 0.000655 0.006163 |
| 0.730192 | 4.98E-06 0.000102 |
| 0.733844 | 1.76E-05 0.00029 |
| 0.463684 | 5.03E-22 1.08E-19 |
| 1.403179 | 1.01E-08 3.59E-07 |
| 1.979793 | 0.001219 0.009386 |
| 1.104143 | 0.009262 0.038119 |
| 0.693594 | 0.008864 0.036831 |
| 0.574306 | 0.000698 0.006174 |
| 1.481326 | 0.011434 0.043477 |
| 1.130291 | 0.007958 0.034753 |
| 0.641802 | 0.001377 0.01016 |
| 1.227637 | 5.75E-07 1.45E-05 |
| 0.801489 | 0.010595 0.041223 |
| 0.584883 | 0.000176 0.002091 |
| 1.367847 | 0.007159 0.033029 |
| 1.360396 | 5.90E-05 0.000743 |

Q9C040 TRIM2 Tripartite S424 0 NA NA NA 4 2.148365 0.009977 0.030573 0 NA NA NA 4

Q7Z2D5 LPPR4 Lipid phos S472 0 NA NA NA 10 1.953729 0.004876 0.017431 0 NA NA NA 10

Q92974 ARHGEF2 Rho guani S696 0 NA NA NA 6 1.994963 0.000976 0.004653 0 NA NA NA 6

Q9UPR5 SLC8A2 Sodium/ca S622 2 1.027896 NA NA 58 1.616705 3.78E-10 7.73E-09 0 NA NA NA 64

P60709 ACTB Actin, cyto S239 1 3.701661 NA NA 26 1.720015 3.71E-08 5.30E-07 1 2.409977 NA NA 25

P68032 ACTC1 Actin, alph S241 1 3.701661 NA NA 26 1.720015 3.71E-08 5.30E-07 1 2.409977 NA NA 25

P37837 TALDO1 Transaldol S237 6 1.590327 0.21907 0.495483 18 1.759107 1.65E-12 5.45E-11 6 1.360642 0.270223 0.427178 18

P10644 PRKAR1A cAMP-dep S83 13 1.191359 0.264282 0.542058 14 1.327466 0.000684 0.003455 10 1.289572 0.148428 0.280758 13

P29966 MARCKS Myristoyla S132 2 1.331986 NA NA 37 1.396675 9.17E-05 0.000625 2 1.276765 NA NA 35

P46821 MAP1B Microtubul S937 36 1.078772 0.342049 0.595189 13 0.741901 0.000183 0.001119 35 1.18533 0.11779 0.239103 13

Q9H3Z4 DNAJC5 DnaJ hom S10 44 1.02198 0.764862 0.89918 34 0.684706 1.26E-07 1.69E-06 45 1.148234 0.015911 0.062461 34

Q01082 SPTBN1 Spectrin b S2138 95 1.062416 0.257149 0.531956 205 1.544188 5.34E-28 5.72E-26 91 1.097474 0.013411 0.054917 200

P46821 MAP1B Microtubul S1265 75 0.794132 0.000508 0.009794 75 1.195919 0.000147 0.000939 73 1.080866 0.27809 0.427485 74

P07197 NEFM NeurofilamS559 82 1.156658 0.034789 0.163466 20 0.66518 5.56E-06 5.30E-05 80 1.077687 0.072336 0.172662 20

P04075 ALDOA Fructose-bS39 43 1.034247 0.610233 0.809423 82 1.306699 6.55E-09 1.12E-07 41 1.068098 0.240106 0.394638 73

O75508 CLDN11 Claudin-11S198 79 1.013144 0.779243 0.902355 18 1.379985 0.000261 0.001558 79 1.065754 0.312992 0.464598 18

P49006 MARCKSLMARCKS- S22 16 1.21645 0.204345 0.475817 30 1.271898 0.002583 0.010355 14 1.064013 0.592866 0.71114 28

P55087 AQP4 Aquaporin S285 75 0.662493 3.74E-15 6.02E-13 33 1.208654 0.000486 0.002692 70 1.063854 0.204471 0.354466 32

P49418 AMPH Amphiphy S262 324 1.068861 0.049302 0.206849 252 1.189295 3.05E-08 4.68E-07 313 1.060331 0.033394 0.104 237

Q9UQM7 CAMK2A Calcium/c S333 38 0.760031 0.000711 0.011548 47 0.877958 0.029503 0.068415 41 1.051234 0.357471 0.502363 47

P46821 MAP1B Microtubul S1208 81 1.127705 0.034932 0.163466 145 1.420099 2.63E-25 2.26E-23 75 1.042084 0.493626 0.635426 140

P35611 ADD1 Alpha-add S465 14 2.584569 0.000252 0.005785 8 1.322133 0.000462 0.002609 13 1.029528 0.927327 0.953393 7

Q7Z6L0 PRRT2 Proline-ric S90 38 0.897448 0.328183 0.585867 71 1.156744 0.000512 0.002779 37 1.0289 0.651547 0.759292 71

P11137 MAP2 Microtubul S1158 26 0.738321 0.004845 0.049684 13 0.891534 0.175908 0.264788 27 1.013886 0.805534 0.874023 13

| 1.551919 | 2.33E-05 0.000193 |
| --- | --- |
| 0.647163 | 0.00032 0.001878 |
| 0.329852 | 6.96E-07 7.47E-06 |
| 1.702911 | 2.85E-12 8.15E-11 |
| 0.576177 | 0.002251 0.009377 |
| 1.453955 | 6.51E-20 2.79E-18 |
| 0.546836 | 1.63E-22 8.72E-21 |
| 1.566381 | 9.80E-32 1.40E-29 |
| 0.707961 | 0.005942 0.01969 |
| 1.430869 | 0.000594 0.003146 |
| 0.818936 | 4.16E-05 0.000298 |
| 0.737014 | 3.67E-05 0.000281 |
| 0.503612 | 4.00E-24 2.86E-22 |
| 1.399389 | 0.000826 0.004119 |
| 1.78671 | 0.001007 0.004747 |
| 1.485554 | 1.32E-11 3.15E-10 |
| 0.713045 | 0.019049 0.049229 |
| 0.50216 | 3.35E-05 0.000266 |
| 3.129088 | 5.43E-07 5.97E-06 |
| 1.431708 | 7.97E-09 1.32E-07 |
| 0.62338 | 0.003177 0.012169 |
| 1.373248 | 1.28E-10 2.74E-09 |
| 1.663475 | 0.007613 0.024191 |
| 0.627813 | 0.000118 0.000792 |
| 1.847097 | 2.51E-07 2.99E-06 |
| 1.903485 | 1.02E-07 1.41E-06 |

P46821 MAP1B Microtubul S1205 37 0.996865 0.963359 0.983769 25

Q16623 STX1A Syntaxin-1 S64 31 0.903711 0.115301 0.338179 8

P08238 HSP90AB Heat shoc S226 20 0.959834 0.663295 0.839935 7

Q01082 SPTBN1 Spectrin b S2128 32 1.124829 0.227139 0.502207 46

P46821 MAP1B Microtubul S1881 39 1.042158 0.573705 0.78508 9

Q8IXJ6 SIRT2 NAD-depe S368 280 0.927132 0.103684 0.335407 123

P61266 STX1B Syntaxin-1 S14 157 0.939945 0.004472 0.047902 65

P29966 MARCKS Myristoyla S101 151 0.919086 0.045191 0.199834 288

Q6U841 SLC4A10 Sodium-dr S89 48 0.946266 0.455808 0.711002 21

O00264 PGRMC1 MembraneS181 40 1.063797 0.472476 0.725266 16

P62258 YWHAE 14-3-3 pro S187 70 0.99755 0.967079 0.983795 22

P78559 MAP1A Microtubul S667 36 1.081546 0.127904 0.353643 18

P07900 HSP90AA Heat shoc S263 70 0.921699 0.113397 0.338179 36

P02686 MBP Myelin basS249 249 1.080591 0.120882 0.33875 44

Q96A00 PPP1R14 Protein ph S128 26 0.830667 0.210186 0.485106 15

Q6NV74 KIAA1211 Uncharact S356 4 0.753869 0.239161 0.504082 56

Q9BQI5 SGIP1 SH3-conta S319 10 0.86962 0.144485 0.380557 6

P46821 MAP1B Microtubul S1016 25 0.987173 0.899211 0.952571 9

O00499 BIN1 Myc box-d S303 4 0.951418 0.905866 0.953335 20

Q08495 DMTN Dematin S92 9 0.690719 0.031879 0.156792 54

P11137 MAP2 Microtubul S629 15 0.566296 0.002762 0.034139 16

P11137 MAP2 Microtubul S654 4 0.619367 0.095557 0.326657 83

P29966 MARCKS Myristoyla S128 4 0.893376 0.477394 0.727788 13

Q9UQM7 CAMK2A Calcium/c S78 6 0.983765 0.889867 0.948929 12

Q96TC7 RMDN3 Regulator S46 6 0.455274 0.063967 0.242773 28

Q15149 PLEC Plectin S4386 8 0.869 0.395866 0.660233 29

31 1.012258 0.872649 0.927312 23

31 1.007927 0.882356 0.931375 8

20 1.006166 0.936246 0.95638 7

27 0.989801 0.919323 0.953393 42

39 0.98891 0.915049 0.953176 9

265 0.974671 0.520435 0.650544 121

150 0.969697 0.191617 0.335861 66

145 0.96777 0.401096 0.550638 243

46 0.963771 0.511003 0.64727 18

40 0.946009 0.24645 0.398176 16

68 0.943441 0.238306 0.393039 22

35 0.942783 0.376805 0.521814 16

70 0.936044 0.155762 0.289011 36

221 0.908365 0.114066 0.23354 45

27 0.896124 0.520435 0.650544 14

2 0.889237 NA NA 51

10 0.877749 0.305034 0.455633 6

25 0.841485 0.069484 0.166964 9

5 0.811499 0.583053 0.702919 18

9 0.778583 0.172347 0.307763 45

15 0.707599 0.027904 0.092045 22

3 0.667509 0.178972 0.317208 91

3 0.651023 0.276991 0.427178 11

6 0.618616 0.035119 0.107624 12

6 0.579741 0.063712 0.158445 28

5 0.389781 0.05242 0.139104 27

**DLB** (occipital n temporal) n AD (occipital n temporal)

Human DLB

Occi ·tal Ten pOI'al Occipla l Temporal

Gene

Phosphor nor

Mean

p-value nor

Mean

p-value

nor

Mean

p-value

nor

Mean

p-value

UniProtiD Full name ylation peptide Rat (Welct1's q-value peptide Ratio (Welch's q-value

site fragments test ) fragments test)


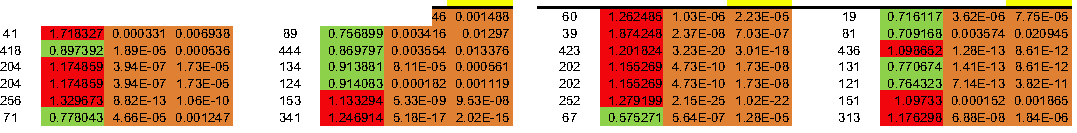


P17600 SYN1 Synapsin-·SJ9

P16949 STMN1 Stathmin S3B

P J1946 YWHAB 14-J-J pro-S1116

P63104 YWHAZ 14-J-3 praS1B4

PJ6!J71 PGM1 PhosphogiS117

P141J6 GFAP Glial fibrii1<:S13

peptide

fragment s

Ratio

(Welch's

test)

pept ide

fragments

Ratio

(Welch's q-value

test)

0 NA NA NA

|  | Human DLB | | | | | | |
| --- | --- | --- | --- | --- | --- | --- | --- |
|  | Occipital | | | |  | Temporal | |
| Phosphor # of p-value  UniProtID Gene Full name ylation peptide Mean (Welch's name site fragments Ratio test) | | | | q-value |  | # of p-value peptide Mean (Welch's fragments Ratio test) | q-value |
| P60201 PLP1 Myelin pro T193 8  P17600 SYN1 Synapsin- S39 41  Q92932 PTPRN2 Receptor-t S360 11  P46821 MAP1B Microtubul S1400 84  P78559 MAP1A Microtubul S1264 8  Q01082 SPTBN1 Spectrin b S2102 38  P35611 ADD1 Alpha-add S358 131  Q9Y2J2 EPB41L3 Band 4.1-l S7 27  Q7Z6L0 PRRT2 Proline-ric S208 131  P09972 ALDOC Fructose-bS39 41  P36871 PGM1 Phosphogl S117 256  P49418 AMPH Amphiphy S549 60  P17252 PRKCA Protein kin S226 26  P07197 NEFM NeurofilamS837 686  Q01082 SPTBN1 Spectrin b S2341 75  P16949 STMN1 Stathmin S38 418  O94811 TPPP Tubulin po S32 100  P31946 YWHAB 14-3-3 pro S186 204  P63104 YWHAZ 14-3-3 pro S184 204  Q16555 DPYSL2 Dihydropy T509 111  P46821 MAP1B Microtubul S1965 10  P46821 MAP1B Microtubul S1817 13  P14136 GFAP Glial fibrillaS13 71  P23528 CFL1 Cofilin-1 S3 40  Q13263 TRIM28 Transcripti S19 5 | | 4.310976 | 0.000132 0.003191 | |  | | |
|  |  | 1.718327 | 0.000331 0.006938 | |  |  |  |
|  |  | 1.488613 | 0.000945 0.014237 | |  |  |  |
|  |  | 1.3198 | 1.77E-06 6.56E-05 | |  |  |  |
|  |  | 1.811004 | 0.000679 0.011548 | |  |  |  |
|  |  | 1.296379 | 0.003368 0.03959 | |  |  |  |
|  |  | 1.328256 | 9.04E-08 6.22E-06 | |  |  |  |
|  |  | 1.278518 | 0.002381 0.030619 | |  |  |  |
|  |  | 1.283901 | 3.07E-07 1.64E-05 | |  |  |  |
|  |  | 1.270525 | 0.003625 0.040631 | |  |  |  |
|  |  | 1.329673 | 8.82E-13 1.06E-10 | |  |  |  |
|  |  | 1.376215 | 3.20E-06 9.63E-05 | |  |  |  |
|  |  | 1.38962 | 0.000639 0.011405 | |  |  |  |
|  |  | 1.304289 | 4.97E-24 2.40E-21 | |  |  |  |
|  |  | 0.664432 | 5.56E-07 2.23E-05 | |  |  |  |
|  |  | 0.897392 | 1.89E-05 0.000536 | |  |  |  |
|  |  | 0.828094 | 0.000267 0.005846 | |  |  |  |
|  |  | 1.174859  1.174859 | 3.94E-07 1.73E-05  3.94E-07 1.73E-05 | |  |  |  |
|  |  | 0.748261 | 2.60E-06 8.77E-05 | |  |  |  |
|  |  | 0.705254  0.585731 | 0.004811 0.049684  0.000415 0.008337 | |  |  |  |
|  |  | 0.778043 | 4.66E-05 0.001247 | |  |  |  |
|  |  | 0.568514 | 0.002212 0.02961 | |  |  |  |
|  |  | 0.35411 | 0.004009 0.043914 | |  |  |  |

| Human AD | | | | | | |
| --- | --- | --- | --- | --- | --- | --- |
| Occipital | | | |  | Temporal | |
| # of p-value peptide Mean (Welch's fragments Ratio test) | | | q-value |  | # of p-value peptide Mean (Welch's fragments Ratio test) | q-value |
| 8  39  12  82  8  38  129  24  136  41  252  60  24  635  75  423  98  202  202  114  9  13  67  41  5 | 6.176634 | 0.001361 0.010352 | |  | | |
|  | 1.874248 | 2.37E-08 7.03E-07 | |  |  |  |
|  | 1.642184 | 0.000841 0.007534 | |  |  |  |
|  | 1.587358 | 5.23E-09 1.77E-07 | |  |  |  |
|  | 1.546928 | 0.003999 0.023745 | |  |  |  |
|  | 1.47613 | 4.96E-14 2.62E-12 | |  |  |  |
|  | 1.404552 | 1.64E-18 1.11E-16 | |  |  |  |
|  | 1.377204 | 3.88E-06 6.82E-05 | |  |  |  |
|  | 1.362348 | 3.84E-24 9.12E-22 | |  |  |  |
|  | 1.29062 | 6.95E-05 0.000944 | |  |  |  |
|  | 1.279199 | 2.15E-25 1.02E-22 | |  |  |  |
|  | 1.262485 | 1.03E-06 2.23E-05 | |  |  |  |
|  | 1.247531 | 3.32E-05 0.000478 | |  |  |  |
|  | 1.221528 | 1.76E-23 2.09E-21 | |  |  |  |
|  | 1.212645 | 0.002753 0.017435 | |  |  |  |
|  | 1.201824 | 3.23E-20 3.01E-18 | |  |  |  |
|  | 1.192191 | 1.24E-05 0.00019 | |  |  |  |
|  | 1.155269  1.155269 | 4.73E-10 1.73E-08  4.73E-10 1.73E-08 | |  |  |  |
|  | 1.130835 | 0.000813 0.007425 | |  |  |  |
|  | 0.683572  0.593443 | 0.001485 0.011025  0.004639 0.025325 | |  |  |  |
|  | 0.575271 | 5.64E-07 1.28E-05 | |  |  |  |
|  | 0.543245 | 1.00E-13 4.77E-12 | |  |  |  |
|  | 0.465483 | 0.00477 0.025747 | |  |  |  |

89 0.756899 0.003416 0.01297

1 0.73652 NA NA

86 1.38577 1.08E-08 1.71E-07

6 0.874332 0.491905 0.582948

22 1.236871 0.015744 0.042748

177 1.19363 5.24E-09 9.53E-08

9 0.785188 0.079952 0.145336

194 1.164047 1.23E-05 0.000103

32 1.220351 0.002545 0.010302

153 1.133294 5.33E-09 9.53E-08

19 0.702035 0.000246 0.001488

7 1.131816 0.04863 0.100785

91 0.944632 0.05858 0.114268

35 0.826999 0.036719 0.080369

444 0.869797 0.003554 0.013376

20 0.933825 0.407803 0.504171

134 0.913881 8.11E-05 0.000561

124 0.914083 0.000182 0.001119

319 0.983617 0.517218 0.602952

3 0.832961 0.395531 0.494703

2 0.578185 NA NA

341 1.246914 5.18E-17 2.02E-15

6 0.493081 0.013443 0.037581

0 NA NA NA

0 NA NA NA

81 0.709168 0.003574 0.020945

1 0.804446 NA NA

89 1.052629 0.191455 0.354731

5 0.737867 0.001848 0.012964

21 0.994303 0.920738 0.945026

168 1.009812 0.732928 0.848849

9 1.229605 0.036357 0.103052

184 0.929378 0.030073 0.089511

31 1.039981 0.440713 0.618089

151 1.09733 0.000152 0.001865

19 0.716117 3.62E-06 7.75E-05

7 1.018045 0.869323 0.923251

97 1.162767 2.32E-06 5.24E-05

35 1.110769 0.180833 0.343984

436 1.098652 1.28E-13 8.61E-12

18 0.935105 0.469599 0.638059

131 0.770674 1.41E-13 8.61E-12

121 0.764323 7.14E-13 3.82E-11

305 1.053995 0.022131 0.074002

3 0.832354 0.237143 0.409264

2 0.857774 NA NA

313 1.176298 6.88E-08 1.84E-06

6 0.964146 0.773234 0.861886

0 NA NA NA

Human DLB

Occipital Temporal

Human AD

Occipital Temporal

UniProtID Gene name

Full name

Phosphor ylation site

| P17600 | SYN1 | Synapsin- S39 | 41 | 1.718327 0.000331 0.006938 | 89 | 0.756899 0.003416 0.01297 |  | 39 | 1.874248 2.37E-08 7.03E-07 | 81 | 0.709168 0.003574 0.020945 |
| --- | --- | --- | --- | --- | --- | --- | --- | --- | --- | --- | --- |
| P16949 | STMN1 | Stathmin S25 | 11 | 1.117224 0.424918 0.67818 | 34 | 1.331706 3.96E-05 0.000288 |  | 10 | 1.867881 0.000252 0.002789 | 54 | 0.955496 0.417019 0.596937 |
| P46821 | MAP1B | Microtubul S1262 | 12 | 1.335355 0.114814 0.338179 | 10 | 1.396955 0.001984 0.008426 |  | 11 | 1.692198 0.002724 0.017435 | 10 | 1.207657 0.054022 0.140131 |
| O60641 | SNAP91 | Clathrin coT312 | 8 | 0.899786 0.648068 0.83521 | 25 | 1.20093 0.002641 0.01049 |  | 8 | 1.68631 0.000314 0.003302 | 24 | 1.154663 0.013935 0.05091 |
| Q13554 | CAMK2B | Calcium/c S367 | 9 | 1.385075 0.031404 0.156792 | 4 | 0.562106 0.000956 0.004653 |  | 9 | 1.683869 1.49E-05 0.000221 | 4 | 0.793376 0.029699 0.089511 |
| P46821 | MAP1B | Microtubul S1400 | 84 | 1.3198 1.77E-06 6.56E-05 | 86 | 1.38577 1.08E-08 1.71E-07 |  | 82 | 1.587358 5.23E-09 1.77E-07 | 89 | 1.052629 0.191455 0.354731 |
| P80723 | BASP1 | Brain acid T196 | 38 | 0.739217 0.05021 0.206849 | 43 | 0.724446 0.000461 0.002609 |  | 38 | 1.57248 2.84E-14 1.69E-12 | 45 | 1.072558 0.48504 0.650946 |
| P07197 | NEFM | NeurofilamS783 | 19 | 1.477364 0.023844 0.124924 | 4 | 0.389673 0.016923 0.045375 |  | 18 | 1.494913 0.002265 0.015151 | 5 | 0.865555 0.518173 0.682258 |
| P29966 | MARCKS | Myristoyla T150 | 39 | 0.73679 0.050173 0.206849 | 40 | 0.579776 0.000647 0.003304 |  | 43 | 1.481222 8.79E-06 0.000139 | 54 | 1.393856 0.000189 0.002127 |
| Q01082 | SPTBN1 | Spectrin b S2102 | 38 | 1.296379 0.003368 0.03959 | 22 | 1.236871 0.015744 0.042748 |  | 38 | 1.47613 4.96E-14 2.62E-12 | 21 | 0.994303 0.920738 0.945026 |
| P35612 | ADD2 | Beta-addu S592 | 57 | 1.296665 0.005886 0.054556 | 73 | 1.125161 0.010134 0.030833 |  | 57 | 1.472853 5.79E-13 2.50E-11 | 67 | 0.957561 0.245595 0.417394 |
| Q02952 | AKAP12 | A-kinase a S283 | 10 | 1.406958 0.067111 0.246 | 21 | 1.675548 1.15E-05 9.85E-05 |  | 11 | 1.428466 0.002586 0.016824 | 21 | 1.282597 0.002364 0.016064 |
| P04406 | GAPDH | GlyceraldeS210 | 20 | 1.365244 0.017146 0.099959 | 28 | 1.274974 4.66E-05 0.000328 |  | 20 | 1.416782 0.000344 0.003475 | 27 | 1.187302 2.17E-05 0.000321 |
| P35612 | ADD2 | Beta-addu S617 | 11 | 1.130149 0.534893 0.759677 | 13 | 1.203113 0.005718 0.019623 |  | 11 | 1.405363 9.44E-05 0.001246 | 13 | 0.854206 0.245755 0.417394 |
| P35611 | ADD1 | Alpha-add S358 | 131 | 1.328256 9.04E-08 6.22E-06 | 177 | 1.19363 5.24E-09 9.53E-08 |  | 129 | 1.404552 1.64E-18 1.11E-16 | 168 | 1.009812 0.732928 0.848849 |
| Q7Z6L0 | PRRT2 | Proline-ric S208 | 131 | 1.283901 3.07E-07 1.64E-05 | 194 | 1.164047 1.23E-05 0.000103 |  | 136 | 1.362348 3.84E-24 9.12E-22 | 184 | 0.929378 0.030073 0.089511 |
| P29966 MARCKS Myristoyla S46 88 1.113989 0.100387 0.335407 180 1.211224 1.92E-07 2.35E-06 86 1.321039 9.62E-08 2.69E-06 170 0.934439 0.007254 0.033029 | | | | | | | | | | | |
| P17677 | GAP43 | Neuromod T107 | 15 | 0.773262 0.173566 0.422518 | 9 | 0.48711 0.000525 0.002813 |  | 15 | 1.311061 0.005542 0.028614 | 10 | 0.783972 0.082861 0.187643 |
| P51608 | MECP2 | Methyl-Cp S80 | 42 | 1.256945 0.023157 0.124019 | 22 | 1.264193 0.019462 0.049996 |  | 41 | 1.30441 5.14E-05 0.000718 | 23 | 1.273084 0.001118 0.00903 |
| P09972 | ALDOC | Fructose-bS39 | 41 | 1.270525 0.003625 0.040631 | 32 | 1.220351 0.002545 0.010302 |  | 41 | 1.29062 6.95E-05 0.000944 | 31 | 1.039981 0.440713 0.618089 |
| P46821 | MAP1B | Microtubul S995 | 26 | 1.107378 0.310613 0.580293 | 17 | 0.618827 0.005833 0.01969 |  | 26 | 1.282506 0.006402 0.03201 | 16 | 0.770824 2.95E-05 0.000421 |
| P36871 | PGM1 | Phosphogl S117 | 256 | 1.329673 8.82E-13 1.06E-10 | 153 | 1.133294 5.33E-09 9.53E-08 |  | 252 | 1.279199 2.15E-25 1.02E-22 | 151 | 1.09733 0.000152 0.001865 |
| P78559 | MAP1A | Microtubul S2022 | 43 | 0.840707 0.010394 0.077076 | 31 | 1.275728 0.000968 0.004653 |  | 42 | 1.279116 0.004166 0.024132 | 31 | 1.116821 0.06443 0.160326 |
| P10636 | MAPT | Microtubul T498 | 23 | 1.132584 0.321506 0.583466 | 47 | 1.406638 2.89E-07 3.31E-06 |  | 23 | 1.273167 0.001098 0.008714 | 47 | 0.911696 0.311499 0.493065 |
|  |  |  |  |  |  |  |  |  |  |  |  |
| P49418 | AMPH | Amphiphy S549 | 60 | 1.376215 3.20E-06 9.63E-05 | 19 | 0.702035 0.000246 0.001488 |  | 60 | 1.262485 1.03E-06 2.23E-05 | 19 |  |
| P07900 | HSP90AA | Heat shoc S231 | 15 | 1.031293 0.772366 0.901405 | 5 | 0.528797 0.001059 0.00494 |  | 15 | 1.259015 0.000927 0.007989 | 5 |  |
| P16949 | STMN1 | Stathmin S38 | 418 | 0.897392 1.89E-05 0.000536 | 444 | 0.869797 0.003554 0.013376 |  | 423 | 1.201824 3.23E-20 3.01E-18 | 436 |  |
| Q8N111 | CEND1 | Cell cycle S87 | 178 | 1.093238 0.006296 0.057255 | 68 | 0.849125 1.63E-07 2.05E-06 |  | 178 | 1.201262 7.05E-24 1.12E-21 | 68 | 0.898863 0.077321 0.178882 |
| Q9C040 | TRIM2 | Tripartite S428 | 54 | 1.112318 0.143272 0.380557 | 85 | 1.261213 2.93E-07 3.31E-06 |  | 53 | 1.168345 0.004329 0.024777 | 80 |  |
| P46821 | MAP1B | Microtubul S1312 | 77 | 1.141216 0.017213 0.099959 | 33 | 0.877086 0.002489 0.010171 |  | 75 | 1.166163 0.000202 0.00229 | 28 |  |
| P31946 | YWHAB | 14-3-3 pro S186 | 204 | 1.174859 3.94E-07 1.73E-05 | 134 | 0.913881 8.11E-05 0.000561 |  | 202 | 1.155269 4.73E-10 1.73E-08 | 131 |  |
| P63104 | YWHAZ | 14-3-3 pro S184 | 204 | 1.174859 3.94E-07 1.73E-05 | 124 | 0.914083 0.000182 0.001119 |  | 202 | 1.155269 4.73E-10 1.73E-08 | 121 |  |
| P35611 | ADD1 | Alpha-add S600 | 30 | 0.917686 0.103058 0.335407 | 16 | 0.874255 0.00247 0.010171 |  | 31 | 1.123324 0.000465 0.004602 | 15 |  |
| P07197 | NEFM | NeurofilamS736 | 690 | 1.064583 0.019411 0.106318 | 177 | 1.360958 1.01E-21 4.83E-20 |  | 687 | 1.097709 1.95E-07 4.64E-06 | 178 |  |
| O00499 | BIN1 | Myc box-d S298 | 166 | 0.929065 0.056464 0.221264 | 334 | 2.082539 1.62E-58 6.96E-56 |  | 150 | 0.886565 0.007147 0.034168 | 320 |  |
| P17677 | GAP43 | Neuromod T181 | 37 | 0.635575 0.015843 0.096665 | 154 | 1.126276 0.000625 0.003272 |  | 33 | 0.729723 0.003257 0.019994 | 138 | 1.034417 0.452286 0.626476 |
| Q6H8Q1 | ABLIM2 | Actin-bindi S294 | 6 | 0.779837 0.276154 0.545328 | 20 | 1.33462 0.002737 0.010674 |  | 5 | 0.699646 0.006556 0.032318 | 16 | 1.14271 0.139089 0.280803 |
| Q16623 | STX1A | Syntaxin-1 S14 | 17 | 0.78383 0.029493 0.15123 | 15 | 0.524775 9.16E-07 9.58E-06 |  | 17 | 0.669192 0.000107 0.001271 | 15 |  |
| Q9UN36 | NDRG2 | Protein NDS332 | 64 | 0.802634 0.005172 0.051021 | 208 | 1.679942 1.36E-22 8.36E-21 |  | 55 | 0.656998 1.10E-07 2.91E-06 | 175 |  |
| P14136 | GFAP | Glial fibrillaS13 | 71 | 0.778043 4.66E-05 0.001247 | 341 | 1.246914 5.18E-17 2.02E-15 |  | 67 | 0.575271 5.64E-07 1.28E-05 | 313 |  |
| P23528 | CFL1 | Cofilin-1 S3 | 40 | 0.568514 0.002212 0.02961 | 6 | 0.493081 0.013443 0.037581 |  | 41 | 0.543245 1.00E-13 4.77E-12 | 6 | 0.964146 0.773234 0.861886 |
| P29966 | MARCKS | Myristoyla S118 | 12 | 0.659674 0.101099 0.335407 | 60 | 1.901219 1.70E-11 3.85E-10 |  | 11 | 0.510048 0.007654 0.035998 | 56 | 0.762605 3.48E-08 9.94E-07 |
| P10636 | MAPT | Microtubul S531 | 8 | 0.956259 0.876781 0.943322 | 56 | 1.425878 5.35E-06 5.22E-05 |  | 8 | 0.41077 0.011642 0.04982 | 52 | 0.843417 0.363933 0.548463 |
| P11137 | MAP2 | Microtubul S833 | 8 | 0.376407 0.013865 0.089103 | 49 | 0.771519 0.018837 0.048976 |  | 8 | 0.233561 0.000942 0.007989 | 50 | 0.895691 0.434968 0.616444 |

# of peptide fragments

Mean

Ratio

p-value (Welch's test)

q-value

# of peptide fragments

Mean

Ratio

p-value (Welch's test)

q-value

# of peptide fragments

Mean

Ratio

p-value (Welch's test)

q-value

# of peptide fragments

Mean

Ratio

p-value (Welch's test)

q-value

Q9H4G0 EPB41L1 Band 4.1-l S678 14 1.318598 0.054454 0.215666 7 0.560815 0.013491 0.037581 12 1.264465 0.000806 0.007425 7 0.900087 0.514148 0.681286

| 0.716117 | 3.62E-06 7.75E-05 |
| --- | --- |
| 0.502082 | 0.004645 0.024165 |
| 1.098652 | 1.28E-13 8.61E-12 |

| 1.152987 | 0.000457 0.004547 |
| --- | --- |
| 0.783697 | 0.003314 0.020265 |
| 0.770674  0.764323 | 1.41E-13 8.61E-12  7.14E-13 3.82E-11 |
| 0.741119 | 0.001212 0.009386 |
| 1.534712 | 1.10E-21 1.57E-19 |
| 1.466685 | 9.11E-25 3.90E-22 |

| 0.518578 | 2.87E-08 8.80E-07 |
| --- | --- |
| 1.185832 | 5.89E-05 0.000743 |
| 1.176298 | 6.88E-08 1.84E-06 |

|  | Human DLB | | | | | | | | |
| --- | --- | --- | --- | --- | --- | --- | --- | --- | --- |
|  | Occipital | | | |  | Temporal | | | |
| Phosphor # of p-value  UniProtID Gene Full name ylation peptide Mean (Welch's name site fragments Ratio test) | | | | q-value |  | # of p-value peptide Mean (Welch's fragments Ratio test) | | | q-value |
| P46821 MAP1B Microtubul S1265 75  P14136 GFAP Glial fibrillaS13 71  P07197 NEFM NeurofilamS837 686  P55087 AQP4 Aquaporin S285 75  P16949 STMN1 Stathmin S38 418  P36871 PGM1 Phosphogl S117 256  Q9UQM7 CAMK2A Calcium/c S333 38  P31946 YWHAB 14-3-3 pro S186 204  P63104 YWHAZ 14-3-3 pro S184 204  P78559 MAP1A Microtubul S1264 8  P49418 AMPH Amphiphy S549 60  P17600 SYN1 Synapsin- S39 41  P61266 STX1B Syntaxin-1 S14 157  P11137 MAP2 Microtubul S629 15  P11137 MAP2 Microtubul S1158 26  P35611 ADD1 Alpha-add S465 14 | | 0.794132 | 0.000508 0.009794 | | 75  341 | | 1.195919 | 0.000147 0.000939 | |
|  |  | 0.778043 | 4.66E-05 0.001247 | |  |  | 1.246914 | 5.18E-17 2.02E-15 | |
|  |  | 1.304289 | 4.97E-24 2.40E-21 | |  | | | | |
|  |  | 0.662493 | 3.74E-15 6.02E-13 | |  |  |  |  |  |
|  |  | 0.897392 | 1.89E-05 0.000536 | |  |  |  |  |  |
|  |  | 1.329673 | 8.82E-13 1.06E-10 | |  |  |  |  |  |
|  |  | 0.760031 | 0.000711 0.011548 | |  |  |  |  |  |
|  |  | 1.174859 | 3.94E-07 1.73E-05 | |  |  |  |  |  |
|  |  | 1.174859 | 3.94E-07 1.73E-05 | |  |  |  |  |  |
|  |  | 1.811004 | 0.000679 0.011548 | |  |  |  |  |  |
|  |  | 1.376215  1.718327 | 3.20E-06 9.63E-05  0.000331 0.006938 | |  |  |  |  |  |
|  |  | 0.939945 | 0.004472 0.047902 | |  |  |  |  |  |
|  |  | 0.566296 | 0.002762 0.034139 | |  |  |  |  |  |
|  |  | 0.738321 | 0.004845 0.049684 | |  |  |  |  |  |
|  |  | 2.584569 | 0.000252 0.005785 | |  |  |  |  |  |

91 0.944632 0.05858 0.114268

| Human AD | | | | | | |
| --- | --- | --- | --- | --- | --- | --- |
| Occipital | | | Temporal | | | |
| # of p-value peptide Mean (Welch's fragments Ratio test) | q-value | # of p-value peptide Mean (Welch's fragments Ratio test) | | | | q-value |
| 73 1.080866 0.27809 0.427485 74  67 0.575271 5.64E-07 1.28E-05 313  635 1.221528 1.76E-23 2.09E-21 97  70 1.063854 0.204471 0.354466 32  423 1.201824 3.23E-20 3.01E-18 436  252 1.279199 2.15E-25 1.02E-22 151  41 1.051234 0.357471 0.502363 47  202 131  202 121  8 5  60 19  39 81  150 0.969697 0.191617 0.335861 66  15 0.707599 0.027904 0.092045 22  27 1.013886 0.805534 0.874023 13  13 1.029528 0.927327 0.953393 7 | | | | 1.234383 | 7.27E-06 0.000141 | |
|  |  |  |  | 1.176298 | 6.88E-08 1.84E-06 | |
|  |  |  |  | 1.162767 | 2.32E-06 5.24E-05 | |
|  |  |  |  | 1.140161 | 0.002262 0.015614 | |
|  |  |  |  | 1.098652 | 1.28E-13 8.61E-12 | |
|  |  |  |  | 1.09733 | 0.000152 0.001865 | |
|  |  |  |  | 0.84548 | 0.00965 0.038599 | |
|  |  |  |  | 0.770674 | 1.41E-13 8.61E-12 | |
|  |  |  |  | 0.764323 | 7.14E-13 3.82E-11 | |
|  |  |  |  | 0.737867 | 0.001848 0.012964 | |
|  |  |  |  | 0.716117  0.709168 | 3.62E-06 7.75E-05  0.003574 0.020945 | |
|  |  |  |  | 0.685814 | 3.03E-14 3.24E-12 | |
|  |  |  |  | 0.641802 | 0.001377 0.01016 | |
|  |  |  |  | 0.593971 | 0.000691 0.006174 | |
|  |  |  |  | 0.152934 | 0.008488 0.035968 | |

33 1.208654 0.000486 0.002692

444 0.869797 0.003554 0.013376

153 1.133294 5.33E-09 9.53E-08

47 0.877958 0.029503 0.068415

| 1.155269 | 4.73E-10 1.73E-08 |
| --- | --- |
| 1.155269 | 4.73E-10 1.73E-08 |
| 1.546928 | 0.003999 0.023745 |
| 1.262485  1.874248 | 1.03E-06 2.23E-05  2.37E-08 7.03E-07 |

134 0.913881 8.11E-05 0.000561

124 0.914083 0.000182 0.001119

6 0.874332 0.491905 0.582948

| 0.702035  0.756899 | 0.000246 0.001488  0.003416 0.01297 |
| --- | --- |
| 0.546836 | 1.63E-22 8.72E-21 |
| 0.62338 | 0.003177 0.012169 |

19

89

65

16

13 0.891534 0.175908 0.264788

8 1.322133 0.000462 0.002609

Human DLB

Occipital Temporal

Human AD

Occipital Temporal

UniProtID Gene name

Full name

Phosphor ylation site

# of peptide fragments

Mean

Ratio

p-value (Welch's test)

q-value

# of peptide fragments

Mean

Ratio

p-value (Welch's test)

q-value

# of peptide fragments

Mean

Ratio

p-value (Welch's test)

q-value

# of peptide fragments

Mean

Ratio

p-value (Welch's test)

q-value

Q9C040 TRIM2 Tripartite S424 0 NA NA NA 4 2.148365 0.009977 0.030573 0 NA NA NA 4 2.014409 0.000339 0.00354

Q96A00 PPP1R14 Protein ph S128 26 0.830667 0.210186 0.485106 15 1.78671 0.001007 0.004747 27 0.896124 0.520435 0.650544 14 1.979793 0.001219 0.009386

P37837 TALDO1 Transaldol S237 6 1.590327 0.21907 0.495483 18 1.759107 1.65E-12 5.45E-11 6 1.360642 0.270223 0.427178 18 1.943769 0.003093 0.020058

O75508 CLDN11 Claudin-11S198 79 1.013144 0.779243 0.902355 18 1.379985 0.000261 0.001558 79 1.065754 0.312992 0.464598 18 1.636194 0.003295 0.020265

P07197 NEFM NeurofilamS736 690 1.064583 0.019411 0.106318 177 1.360958 1.01E-21 4.83E-20 687 1.097709 1.95E-07 4.64E-06 178 1.534712 1.10E-21 1.57E-19

O00499 BIN1 Myc box-d S303 4 0.951418 0.905866 0.953335 20 3.129088 5.43E-07 5.97E-06 5 0.811499 0.583053 0.702919 18 1.481326 0.011434 0.043477

Q8IXJ6 SIRT2 NAD-depe S368 280 0.927132 0.103684 0.335407 123 1.453955 6.51E-20 2.79E-18 265 0.974671 0.520435 0.650544 121 1.477753 1.37E-10 5.84E-09

O00499 BIN1 Myc box-d S298 166 0.929065 0.056464 0.221264 334 2.082539 1.62E-58 6.96E-56 150 0.886565 0.007147 0.034168 320 1.466685 9.11E-25 3.90E-22

P02686 MBP Myelin basS249 249 1.080591 0.120882 0.33875 44 1.399389 0.000826 0.004119 221 0.908365 0.114066 0.23354 45 1.403179 1.01E-08 3.59E-07

Q7Z2D5 LPPR4 Lipid phos S472 0 NA NA NA 10 1.953729 0.004876 0.017431 0 NA NA NA 10 1.39923 0.000188 0.002127

P29966 MARCKS Myristoyla T150 39 0.73679 0.050173 0.206849 40 0.579776 0.000647 0.003304 43 1.481222 8.79E-06 0.000139 54 1.393856 0.000189 0.002127

Q96TC7 RMDN3 Regulator S46 6 0.455274 0.063967 0.242773 28 1.847097 2.51E-07 2.99E-06 6 0.579741 0.063712 0.158445 28 1.367847 0.007159 0.033029

Q15149 PLEC Plectin S4386 8 0.869 0.395866 0.660233 29 1.903485 1.02E-07 1.41E-06 5 0.389781 0.05242 0.139104 27 1.360396 5.90E-05 0.000743

| 1.416782 | 0.000344 0.003475 |
| --- | --- |
| 0.656998 | 1.10E-07 2.91E-06 |
| 0.575271 | 5.64E-07 1.28E-05 |

| P29966 MARCKS Myristoyla S132 2  Q92974 ARHGEF2 Rho guani S696 0 | | | 1.331986 NA NA | 37 | 1.396675 9.17E-05 0.000625 | 2 | 1.276765 NA NA | 35 | 1.323532 1.86E-05 0.000295  1.315897 0.00373 0.020994  1.286313 0.01043 0.040954  1.282597 0.002364 0.016064 |
| --- | --- | --- | --- | --- | --- | --- | --- | --- | --- |
|  |  |  | NA NA NA | 6 | 1.994963 0.000976 0.004653 | 0 | NA NA NA | 6 |  |
| P46821 | MAP1B Microtubul S1205 | 37 | 0.996865 0.963359 0.983769 | 25 | 1.551919 2.33E-05 0.000193 | 31 | 1.012258 0.872649 0.927312 | 23 |  |
| Q02952 | AKAP12 A-kinase a S283 | 10 | 1.406958 0.067111 0.246 | 21 | 1.675548 1.15E-05 9.85E-05 | 11 | 1.428466 0.002586 0.016824 | 21 |  |
| P51608 | MECP2 Methyl-Cp S80 | 42 | 1.256945 0.023157 0.124019 | 22 | 1.264193 0.019462 0.049996 | 41 | 1.30441 5.14E-05 0.000718 | 23 | 1.273084 0.001118 0.00903 |
| P46821 | MAP1B Microtubul S1265 | 75 | 0.794132 0.000508 0.009794 | 75 | 1.195919 0.000147 0.000939 | 73 | 1.080866 0.27809 0.427485 | 74 | 1.234383 7.27E-06 0.000141 |
| P46821 | MAP1B Microtubul S1208 | 81 | 1.127705 0.034932 0.163466 | 145 | 1.420099 2.63E-25 2.26E-23 | 75 | 1.042084 0.493626 0.635426 | 140 | 1.231366 2.88E-08 8.80E-07 |
| P11137 | MAP2 Microtubul S654 | 4 | 0.619367 0.095557 0.326657 | 83 | 1.373248 1.28E-10 2.74E-09 | 3 | 0.667509 0.178972 0.317208 | 91 | 1.227637 5.75E-07 1.45E-05 |
| Q01082 | SPTBN1 Spectrin b S2128 | 32 | 1.124829 0.227139 0.502207 | 46 | 1.702911 2.85E-12 8.15E-11 | 27 | 0.989801 0.919323 0.953393 | 42 | 1.209975 0.000662 0.006163 |
| P49006 | MARCKSLMARCKS- S22 | 16 | 1.21645 0.204345 0.475817 | 30 | 1.271898 0.002583 0.010355 | 14 | 1.064013 0.592866 0.71114 | 28 | 1.200343 0.004743 0.024165 |
| P04406 | GAPDH GlyceraldeS210 | 20 | 1.365244 0.017146 0.099959 | 28 | 1.274974 4.66E-05 0.000328 | 20 |  | 27 | 1.187302 2.17E-05 0.000321 |
| Q9UN36 | NDRG2 Protein NDS332 | 64 | 0.802634 0.005172 0.051021 | 208 | 1.679942 1.36E-22 8.36E-21 | 55 |  | 175 | 1.185832 5.89E-05 0.000743 |
| P14136 | GFAP Glial fibrillaS13 | 71 | 0.778043 4.66E-05 0.001247 | 341 | 1.246914 5.18E-17 2.02E-15 | 67 |  | 313 | 1.176298 6.88E-08 1.84E-06 |
| P10644 | PRKAR1A cAMP-dep S83 | 13 | 1.191359 0.264282 0.542058 | 14 | 1.327466 0.000684 0.003455 | 10 | 1.289572 0.148428 0.280758 | 13 | 1.174193 0.009544 0.038599 |
| P60709 | ACTB Actin, cyto S239 | 1 | 3.701661 NA NA | 26 | 1.720015 3.71E-08 5.30E-07 | 1 | 2.409977 NA NA | 25 | 1.158294 0.007331 0.033029 |
| P68032 | ACTC1 Actin, alph S241 | 1 | 3.701661 NA NA | 26 | 1.720015 3.71E-08 5.30E-07 | 1 | 2.409977 NA NA | 25 | 1.158294 0.007331 0.033029 |
| Q9C040 | TRIM2 Tripartite S428 | 54 | 1.112318 0.143272 0.380557 | 85 | 1.261213 2.93E-07 3.31E-06 | 53 | 1.168345 0.004329 0.024777 | 80 | 1.152987 0.000457 0.004547 |
| Q7Z6L0 | PRRT2 Proline-ric S90 | 38 | 0.897448 0.328183 0.585867 | 71 | 1.156744 0.000512 0.002779 | 37 | 1.0289 0.651547 0.759292 | 71 | 1.148606 0.004348 0.023262 |
| P55087 | AQP4 Aquaporin S285 | 75 | 0.662493 3.74E-15 6.02E-13 | 33 | 1.208654 0.000486 0.002692 | 70 | 1.063854 0.204471 0.354466 | 32 | 1.140161 0.002262 0.015614 |
| Q08495 | DMTN Dematin S92 | 9 | 0.690719 0.031879 0.156792 | 54 | 1.431708 7.97E-09 1.32E-07 | 9 | 0.778583 0.172347 0.307763 | 45 | 1.130291 0.007958 0.034753 |
| Q01082 | SPTBN1 Spectrin b S2138 | 95 | 1.062416 0.257149 0.531956 | 205 | 1.544188 5.34E-28 5.72E-26 | 91 | 1.097474 0.013411 0.054917 | 200 | 1.122026 1.93E-06 4.60E-05 |
| Q9UPR5 | SLC8A2 Sodium/ca S622 | 2 | 1.027896 NA NA | 58 | 1.616705 3.78E-10 7.73E-09 | 0 | NA NA NA | 64 | 1.118111 0.00367 0.020945 |
| Q6NV74 | KIAA1211 Uncharact S356 | 4 | 0.753869 0.239161 0.504082 | 56 | 1.485554 1.32E-11 3.15E-10 | 2 | 0.889237 NA NA | 51 | 1.104143 0.009262 0.038119 |
| P16949 | STMN1 Stathmin S38 | 418 | 0.897392 1.89E-05 0.000536 | 444 | 0.869797 0.003554 0.013376 | 423 | 1.201824 3.23E-20 3.01E-18 | 436 | 1.098652 1.28E-13 8.61E-12 |
| P36871 | PGM1 Phosphogl S117 | 256 | 1.329673 8.82E-13 1.06E-10 | 153 | 1.133294 5.33E-09 9.53E-08 | 252 | 1.279199 2.15E-25 1.02E-22 | 151 | 1.09733 0.000152 0.001865 |
| P04075 | ALDOA Fructose-bS39 | 43 | 1.034247 0.610233 0.809423 | 82 | 1.306699 6.55E-09 1.12E-07 | 41 | 1.068098 0.240106 0.394638 | 73 | 1.088428 0.013134 0.048462 |
| P29966 | MARCKS Myristoyla S46 | 88 | 1.113989 0.100387 0.335407 | 180 | 1.211224 1.92E-07 2.35E-06 | 86 | 1.321039 9.62E-08 2.69E-06 | 170 | 0.934439 0.007254 0.033029 |
| P29966 | MARCKS Myristoyla S101 | 151 | 0.919086 0.045191 0.199834 | 288 | 1.566381 9.80E-32 1.40E-29 | 145 | 0.96777 0.401096 0.550638 | 243 | 0.880573 4.58E-09 1.78E-07 |

P49418 AMPH Amphiphy S262 324 1.068861 0.049302 0.206849 252 1.189295 3.05E-08 4.68E-07 313 1.060331 0.033394 0.104 237 0.852442 6.91E-11 3.29E-09

P07197 NEFM NeurofilamS559 82 1.156658 0.034789 0.163466 20 0.66518 5.56E-06 5.30E-05 80 1.077687 0.072336 0.172662 20 0.838198 0.000224 0.002399

P29966 MARCKS Myristoyla S128 4 0.893376 0.477394 0.727788 13 1.663475 0.007613 0.024191 3 0.651023 0.276991 0.427178 11 0.801489 0.010595 0.041223

| 1.166163 | 0.000202 0.00229 |
| --- | --- |
| 1.282506 | 0.006402 0.03201 |
| 1.155269 | 4.73E-10 1.73E-08 |

P46821 MAP1B Microtubul S1312 77 1.141216 0.017213 0.099959 33 0.877086 0.002489 0.010171 75

P46821 MAP1B Microtubul S995 26 1.107378 0.310613 0.580293 17 0.618827 0.005833 0.01969 26

P31946 YWHAB 14-3-3 pro S186 204 1.174859 3.94E-07 1.73E-05 134 0.913881 8.11E-05 0.000561 202

28 0.783697 0.003314 0.020265

16 0.770824 2.95E-05 0.000421

131 0.770674 1.41E-13 8.61E-12

P46821 MAP1B Microtubul S937 36 1.078772 0.342049 0.595189 13 0.741901 0.000183 0.001119 35 1.18533 0.11779 0.239103 13 0.770591 0.00366 0.020945

| P63104 YWHAZ 14-3-3 pro S184  P29966 MARCKS Myristoyla S118  Q9H3Z4 DNAJC5 DnaJ hom S10  Q6U841 SLC4A10 Sodium-dr S89 | | | 204 | 1.174859 3.94E-07 1.73E-05 | 124 | 0.914083 0.000182 0.001119 | 202 | 1.155269 4.73E-10 1.73E-08 | 121 | 0.764323 7.14E-13 3.82E-11 |
| --- | --- | --- | --- | --- | --- | --- | --- | --- | --- | --- |
|  |  |  | 12 | 0.659674 0.101099 0.335407 | 60 | 1.901219 1.70E-11 3.85E-10 | 11 | 0.510048 0.007654 0.035998 | 56 | 0.762605 3.48E-08 9.94E-07 |
|  |  |  | 44 | 1.02198 0.764862 0.89918 | 34 | 0.684706 1.26E-07 1.69E-06 | 45 | 1.148234 0.015911 0.062461 | 34 | 0.760619 0.001228 0.009386 |
|  |  |  | 48 | 0.946266 0.455808 0.711002 | 21 | 0.707961 0.005942 0.01969 | 46 | 0.963771 0.511003 0.64727 | 18 | 0.754464 0.008595 0.036066 |
| P35611 | ADD1 | Alpha-add S600 | 30 | 0.917686 0.103058 0.335407 | 16 | 0.874255 0.00247 0.010171 | 31 | 1.123324 0.000465 0.004602 | 15 | 0.741119 0.001212 0.009386 |
| Q16623 | STX1A | Syntaxin-1 S64 | 31 | 0.903711 0.115301 0.338179 | 8 | 0.647163 0.00032 0.001878 | 31 | 1.007927 0.882356 0.931375 | 8 | 0.740102 0.001536 0.010954 |
| P78559 | MAP1A | Microtubul S667 | 36 | 1.081546 0.127904 0.353643 | 18 | 0.737014 3.67E-05 0.000281 | 35 | 0.942783 0.376805 0.521814 | 16 | 0.733844 1.76E-05 0.00029 |
| P62258 | YWHAE | 14-3-3 pro S187 | 70 | 0.99755 0.967079 0.983795 | 22 | 0.818936 4.16E-05 0.000298 | 68 | 0.943441 0.238306 0.393039 | 22 | 0.730192 4.98E-06 0.000102 |
| P49418 | AMPH | Amphiphy S549 | 60 | 1.376215 3.20E-06 9.63E-05 | 19 | 0.702035 0.000246 0.001488 | 60 | 1.262485 1.03E-06 2.23E-05 | 19 | 0.716117 3.62E-06 7.75E-05 |
| P17600 | SYN1 | Synapsin- S39 | 41 | 1.718327 0.000331 0.006938 | 89 | 0.756899 0.003416 0.01297 | 39 | 1.874248 2.37E-08 7.03E-07 | 81 | 0.709168 0.003574 0.020945 |
| P46821 | MAP1B | Microtubul S1881 | 39 | 1.042158 0.573705 0.78508 | 9 | 0.576177 0.002251 0.009377 | 39 | 0.98891 0.915049 0.953176 | 9 | 0.701072 0.011703 0.043939 |
| O00264 PGRMC1 MembraneS181 40 1.063797 0.472476 0.725266 16 1.430869 0.000594 0.003146 40 0.946009 0.24645 0.398176 16 0.694455 0.000655 0.006163 | | | | | | | | | | |
|  |  |  |  |  |  |  |  |  |  |  |
| P61266 | STX1B | Syntaxin-1 S14 | 157 | 0.939945 0.004472 0.047902 | 65 | 0.546836 1.63E-22 8.72E-21 | 150 | 0.969697 0.191617 0.335861 | 66 | 0.685814 3.03E-14 3.24E-12 |
| P11137 | MAP2 | Microtubul S629 | 15 | 0.566296 0.002762 0.034139 | 16 | 0.62338 0.003177 0.012169 | 15 | 0.707599 0.027904 0.092045 | 22 | 0.641802 0.001377 0.01016 |
| Q9UQM7 | CAMK2A | Calcium/c S78 | 6 | 0.983765 0.889867 0.948929 | 12 | 0.627813 0.000118 0.000792 | 6 | 0.618616 0.035119 0.107624 | 12 | 0.584883 0.000176 0.002091 |
| P46821 | MAP1B | Microtubul S1016 | 25 | 0.987173 0.899211 0.952571 | 9 | 0.50216 3.35E-05 0.000266 | 25 | 0.841485 0.069484 0.166964 | 9 | 0.574306 0.000698 0.006174 |

Q9BQI5 SGIP1 SH3-conta S319 10 0.86962 0.144485 0.380557 6 0.713045 0.019049 0.049229 10 0.877749 0.305034 0.455633 6 0.693594 0.008864 0.036831

| Q16623 STX1A Syntaxin-1 S14 17 0.78383 0.029493 0.15123 15 0.524775 9.16E-07 9.58E-06 | | | | | |  |  |  | 0.518578 2.87E-08 8.80E-07 |
| --- | --- | --- | --- | --- | --- | --- | --- | --- | --- |
|  |  |  |  |  |  |  |  |  |  |
|  |  |  |  |  |  | 17 | 0.669192 0.000107 0.001271 | 15 |  |
| P07900 | HSP90AA Heat shoc S231 | 15 | 1.031293 0.772366 0.901405 | 5 | 0.528797 0.001059 0.00494 | 15 | 1.259015 0.000927 0.007989 | 5 | 0.502082 0.004645 0.024165 |
| P07900 | HSP90AA Heat shoc S263 | 70 | 0.921699 0.113397 0.338179 | 36 | 0.503612 4.00E-24 2.86E-22 | 70 | 0.936044 0.155762 0.289011 | 36 | 0.463684 5.03E-22 1.08E-19 |
| P08238 | HSP90AB Heat shoc S226 | 20 | 0.959834 0.663295 0.839935 | 7 | 0.329852 6.96E-07 7.47E-06 | 20 | 1.006166 0.936246 0.95638 | 7 | 0.266769 1.05E-05 0.000195 |
| P35611 | ADD1 Alpha-add S465 | 14 | 2.584569 0.000252 0.005785 | 8 | 1.322133 0.000462 0.002609 | 13 | 1.029528 0.927327 0.953393 | 7 | 0.152934 0.008488 0.035968 |
