# Supplementary figures and images for: Ser46-Phosphorylated MARCKS Is a Marker of Neurite Degeneration at the Pre-aggregation Stage in PD/DLB Pathology
Source: eNeuro. 2018 Sep 4;5(4):ENEURO.0217-18.2018. doi: 10.1523/ENEURO.0217-18.2018 (PMC6140116; doi:10.1523/ENEURO.0217-18.2018)

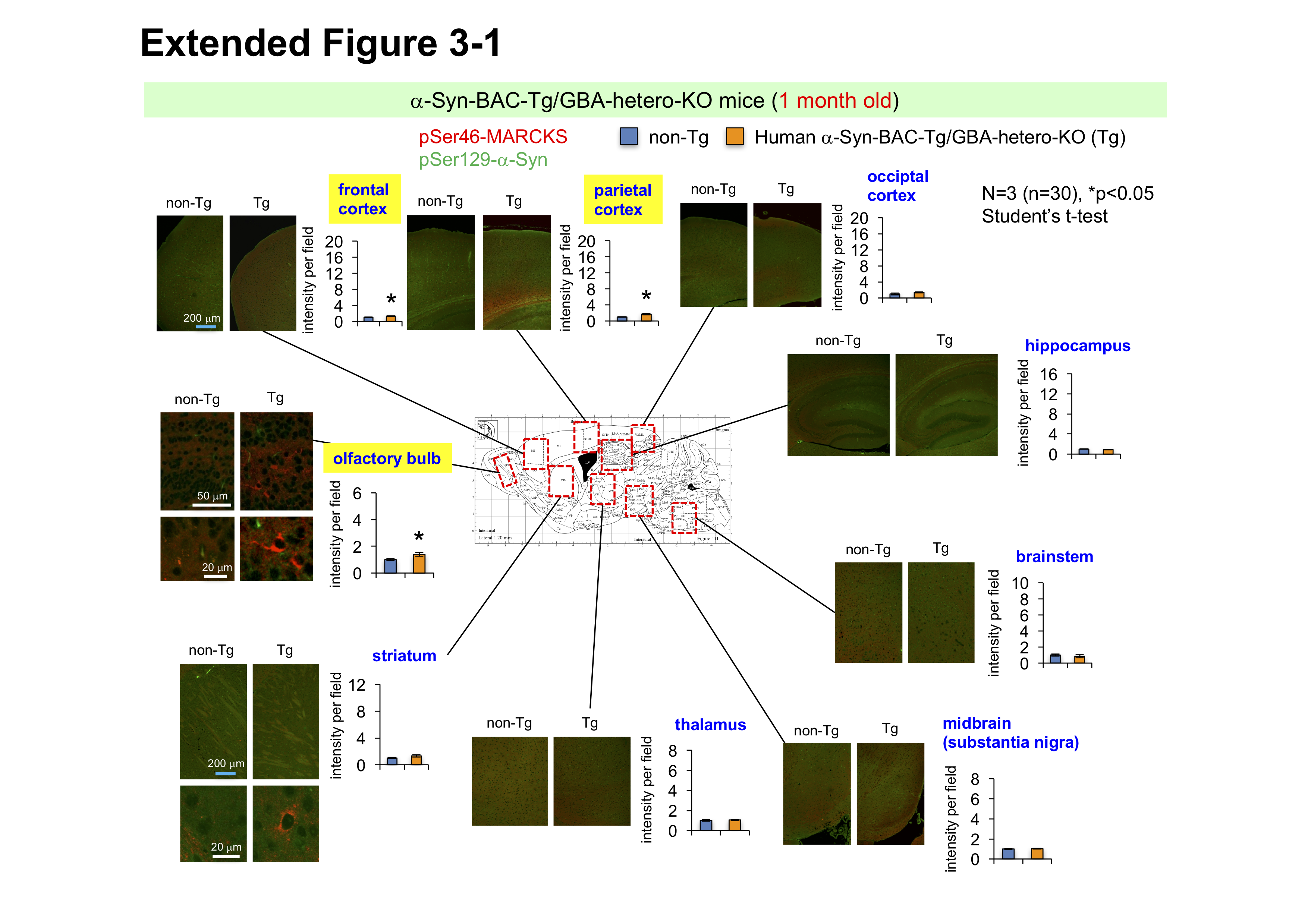

Supplement: Figure 3-1 — pSer46-MARCKS in human α-Syn-BAC-Tg/GBA-hetero-KO mice at 1 month of age. pSer46-MARCKS and pSer129-α-Syn were costained in human normal α-Syn-BAC-Tg/glucocerebrosidase (GBA)-hetero-KO mice at 1 month of age (3 males in each group). Signal intensities were significantly higher in yellow-marked areas. Download Figure 3-1, TIF file. [file sup_enu-eN-NWR-0217-18-s02.tif]

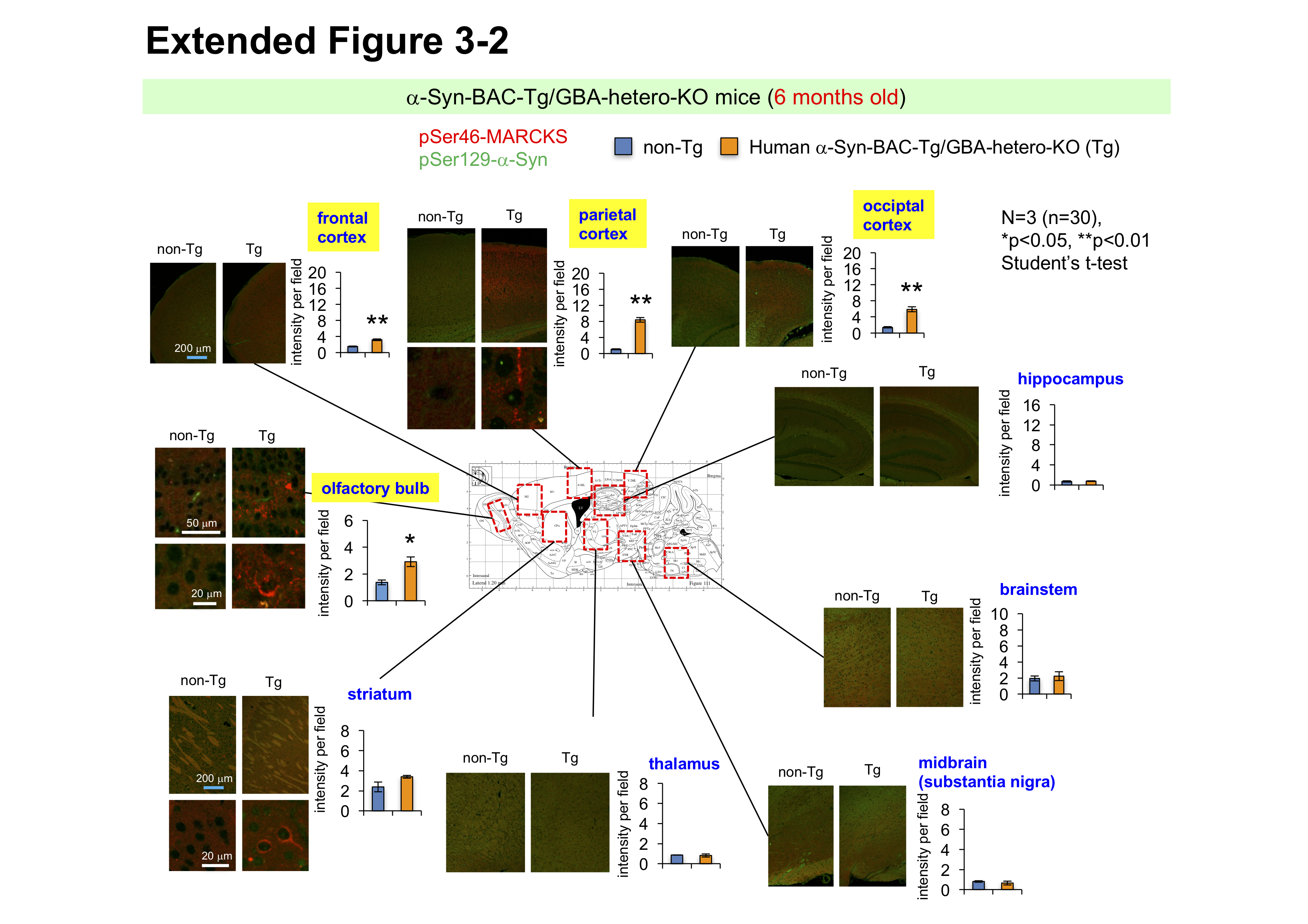

Supplement: Figure 3-2 — pSer46-MARCKS in human α-Syn-BAC-Tg/GBA-hetero-KO mice at 6 months of age. pSer46-MARCKS and pSer129-α-Syn were costained in human normal α-Syn-BAC-Tg/glucocerebrosidase (GBA)-hetero-KO mice at 6 months of age (3 males in each group). Signal intensities were significantly higher in yellow-marked areas. Download Figure 3-2, TIF file. [file sup_enu-eN-NWR-0217-18-s03.tif]

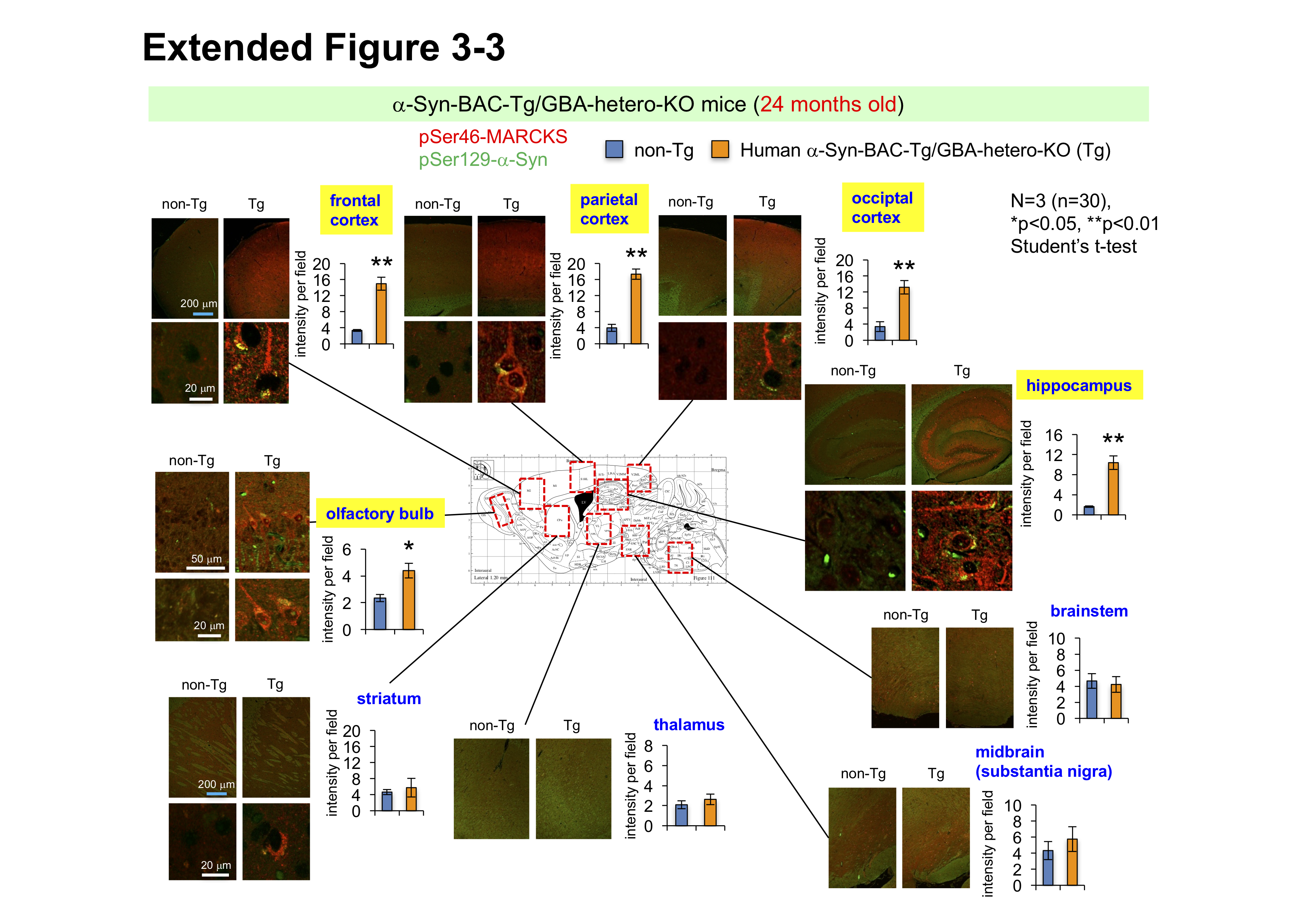

Supplement: Figure 3-3 — pSer46-MARCKS in human α-Syn-BAC-Tg/GBA-hetero-KO mice at 24 months of age. pSer46-MARCKS and pSer129-α-Syn were costained in human normal α-Syn-BAC-Tg/glucocerebrosidase (GBA)-hetero-KO mice at 24 months of age (3 males in each group). Signal intensities were significantly higher in yellow-marked areas. Download Figure 3-3, TIF file. [file sup_enu-eN-NWR-0217-18-s04.tif]

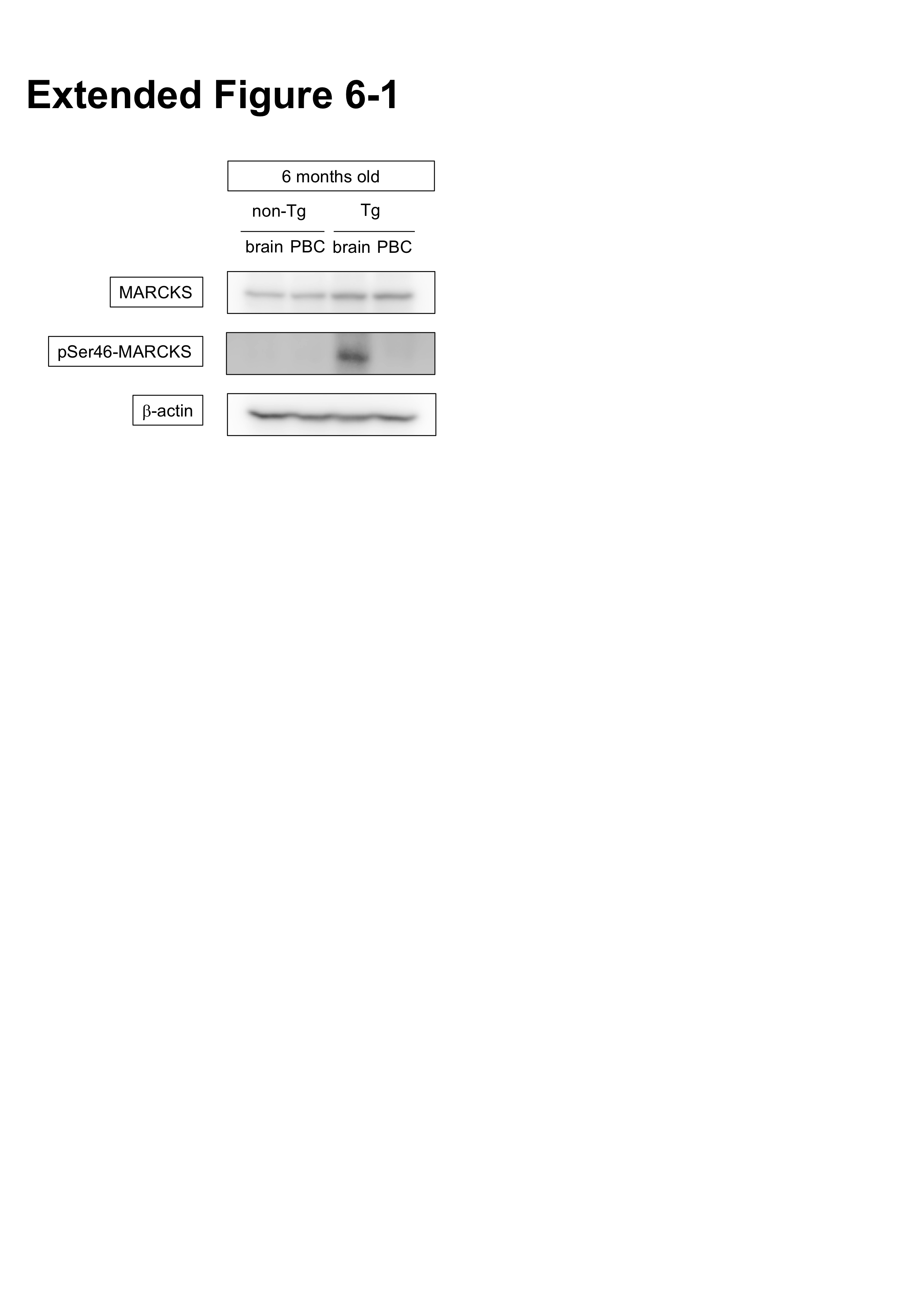

Supplement: Figure 6-1 — Protein levels of pSer46-MARCKS were compared between peripheral blood cells (PBC) and whole cerebral cortex (brain) of α-Syn-BAC-Tg/GBA-hetero-KO (Tg) or the nontransgenic sibling control (non-Tg) mice at 6 months of age. Download Figure 6-1, TIF file. [file sup_enu-eN-NWR-0217-18-s05.tif]
